# Effect of Formulation and Fermentation on Protein Structure, Nutritional Characteristics and Techno-Functional Properties of Hemp–Oat Press Cake Matrices

**DOI:** 10.3390/foods15183357

**Published:** 2026-09-21

**Authors:** Erika Keiko Martinez Vargas, Alvija Šalaševičienė, Per Ertbjerg

**Affiliations:** 1 Food Institute, Kaunas University of Technology, LT-50254 Kaunas, Lithuania; alvija.salaseviciene@ktu.lt; 2 Department of Food and Nutrition, University of Helsinki, FI-00014 Helsinki, Finland; per.ertbjerg@helsinki.fi

**Keywords:** plant-based foods, press cakes, lactic acid fermentation, protein structure, antinutritional factors, sustainability, plant protein, techno-functional properties

## Abstract

The valorization of plant-based press cakes as sustainable food ingredients requires understanding how formulation and processing affect their properties. This study investigated mixed formulations of hemp press cakes and oat press cakes subjected to pasteurization and lactic acid fermentation using a single independently prepared batch per formulation × treatment condition. A multi-analytical approach was applied, including protein solubility, SDS-PAGE, particle size analysis, evaluation of amino acids and biogenic amines, protein oxidation, phytic acid content, and techno-functional properties (water- and oil-holding capacities). The relative importance of formulation and processing was parameter-dependent. Formulation was the main source of variation in total amino acid composition, phytic acid levels, biogenic amine accumulation, and protein solubility under multi-bond disruption conditions. Processing was associated with protein aggregation, confirmed by SDS-PAGE and particle size increases, whereas fermentation was associated with partial solubility restoration, possibly through pH-mediated changes and structural rearrangements. Nutritional parameters showed formulation–processing interactions, affecting amino acid release, biogenic amine formation, protein oxidation and phytic acid levels, with the latter two remaining relatively high across all conditions. Water-holding capacity varied mainly with formulation, while oil-holding capacity varied with both factors and showed strong interaction patterns. Among the formulations studied, the 75:25 hemp-to-oat ratio represented the most balanced option, offering nutritional and functional improvements over pure hemp press cakes without substantially compromising protein content. Overall, these findings suggest that hemp–oat press cake matrices behave as interaction-sensitive materials and that combined formulation and fermentation strategies may be used to tailor functional and nutritional properties for food applications.

## 1. Introduction

The increasing adoption of vegan, vegetarian, and flexitarian diets is motivated by various factors, such as animal ethics, environmental concerns, nutrition, and health risks [1,2,3]. While many people still appreciate meat for its sensory properties or cultural significance in local cuisines [4], plant raw materials are increasingly being used to produce meat-like products or analogues to replace meat as an ingredient [5]. Moreover, there is a growing trend in the plant-based industry to pair novel structuring techniques, such as 3D-printing and wet spinning, with well-known biochemical processing methods, like fermentation, to offer nutritious and sustainable options that are attractive to consumers [3,6].

Press cakes, byproducts of the oil extraction industry, are emerging as valuable raw materials for food production due to their high content of protein and bioactive compounds [7,8]. Hemp press cakes (HPCs), derived from cold pressing hemp seeds for oil, are of particular interest among oilseed press cakes because of their relatively high protein content (20–40%) [9,10,11], high levels of essential amino acids and arginine [12], and the expanding hemp oil industry in Lithuania, which increasingly generates volumes of this byproduct with limited valorization in human food applications [13].

Despite their potential, press cakes are often underutilized, with many being discarded or limited to low-value applications such as animal feed [8]. The nonprotein fraction of press cakes, which includes dietary fiber, minerals, and bioactive compounds, remains largely underutilized in industrial processes aimed at producing protein concentrates and isolates [14]. Additional processing steps such as precipitation, filtration and concentration also consume significant energy and water resources, raising concerns about sustainability. Moreover, plant protein solubility remains a challenging limitation for most traditional extraction methods [15,16]. A shift toward minimal processing and the direct incorporation of press cakes into food formulations could reduce waste and energy use while enhancing the overall sustainability of plant-based food systems.

The use of press cakes as protein sources is not without challenges. Plant proteins are generally considered of lower quality than animal proteins due to limitations in their essential amino acid profiles and reduced digestibility [17]. Additionally, plant matrices contain antinutritional factors such as phytic acid, tannins, saponins, and lectins, which interfere with nutrient absorption and protein digestibility by inhibiting digestive enzymes [18].

Fermentation is a promising strategy to improve the nutritional and functional properties of press cakes. Fermentation can enhance protein digestibility by partially denaturing storage proteins, increasing protein solubility, and improving amino acid availability [19,20]. Additionally, fermentation has been shown to significantly reduce antinutritional factors such as phytic acid in cereals and pseudocereals [21,22] and could have similar effects on press cakes. Beyond nutritional improvements, fermentation may also enhance the techno-functional properties of press cakes, such as WHC, emulsification, and gelling properties, which are critical for their application in food formulations [23].

While some individual press cakes have been studied for their fermentation potential, the effects of fermentation on combined press cake matrices remain largely unexplored. Hemp press cake was selected as the primary matrix in this study due to its notable protein and fiber content [12]. However, its low carbohydrate content (~3.7 g/100 g according to Nakov et al. [24]) limits the availability of fermentable substrate for lactic acid bacteria, which rely on water-soluble carbohydrates as their primary carbon source. Oat press cake was incorporated as a functional modifier to address this limitation: its residual starch content can serve as an additional fermentable carbohydrate source, while also contributing to the modulation of expansion and texture in further processing steps (i.e., high-moisture extrusion processing) [25]. Combining these two press cakes was intended to exploit their compositional complementarity, as blending plant-derived ingredients with distinct nutritional and functional profiles has been proposed as a strategy to mutually compensate for individual limitations [26]. Fermentation of this combined matrix may further modify its nutritional value and techno-functional properties, supporting its potential application in plant-based food product development.

The current study aims to investigate the effect of lactic acid fermentation on the protein structure, nutritional characteristics, and techno-functional parameters of combined press cake matrices. Specifically, the study evaluates changes in protein oxidation, degradation and bond profiles, amino acid and biogenic amine profiles and phytic acid content. Additionally, the research examines WHC, oil-holding capacity (OHC), and particle size, with the goal of developing sustainable and nutritious plant-based food formulations. We hypothesize that lactic acid fermentation of combined hemp–oat press cake matrices will modulate their protein structure, nutritional characteristics, and techno-functional properties in a formulation-dependent manner, with expected reductions in antinutritional factor content and protein oxidation and improvements in protein solubility, WHC, and OHC relative to unprocessed materials.

## 2. Materials and Methods

### 2.1. Materials

#### 2.1.1. Plant-Based Press Cake Raw Material Preparation

Organic hemp press cake (HPC) granules were received from Allive Europe UAB, Voškoniai, Lithuania. The material originated from cold-pressed organic hemp seeds (maximum outflow temperature 40 °C) and was subsequently pressed into granules. No chemical treatments were applied during processing. At the KTU Food Institute laboratory, the granules were ground in two stages to ensure sample particle size uniformity. Coarse grinding was done with a manual meat grinder, while finer grinding was achieved with a universal dry product mill (MLVS-M100, Mažeikiai, Lithuania). The ground material was sieved using a stainless steel 500 mic sieve (Glenammer, Scotland, UK) and separated into two fractions with a particle size >500 µm and <500 µm mesh size; the smaller particle size fraction was used for all further experiments.

Oat press cake (OPC) was produced on-site from commercial oat (Pranciškaus malūnas, Kaunas, Lithuania). Oat flakes were blended with distilled water for 20 s and pressed through a cheesecloth. The oat press cake was oven-dried at 45 °C for 12 h, mechanically ground with a coffee grinder, and sieved (mesh size 500 µm).

#### 2.1.2. Plant-Based Press Cake Formulations: H100, H75:O25 and H50:O50

Two formulations of combined plant-based press cake matrices, composed of HPC and OPC, were prepared for fermentation experiments at HPC:OPC blend ratios of 75:25 (H75:O25) and 50:50 (H50:O50). These ratios were selected to represent a gradient of OPC inclusion: the 75:25 ratio maintains HPC as the dominant fraction, preserving its protein contribution while introducing a moderate amount of OPC to modulate fermentation substrate availability and functional properties, whereas the 50:50 ratio represents the upper inclusion level before protein content is compromised by the lower protein content of OPC. A third formulation of pure HPC (H100) was also included as a baseline reference.

#### 2.1.3. Plant-Based Press Cake Treatments: Raw (RAW), Pasteurized (PAS) and Fermented (FER)

For the raw samples (RAW), 500 g of each press cake formulation (H100, H75:O25 and H50:O50) was left untreated, transferred to plastic bags, vacuum sealed and stored at 4 °C until analysis.

For the pasteurized samples (PAS), 500 g of each press cake formulation (H100, H75:O25 and H50:O50) was mixed with 1450 mL of distilled water in a 2 L glass jar. The mixtures were pasteurized as follows: heating was carried out to 80 °C using a double boiler over an induction hot plate. The temperature was maintained for 10 min and controlled using a thermometer. The mixture was agitated every minute using a sterile metal table knife. After 10 min, the mixture was immediately cooled to 40 °C by submerging the closed jar in an ice water bath. The resulting mixtures were spread on a tin tray and oven-dried at 60 °C for 24 h. The dried samples were transferred to plastic bags, vacuum sealed and stored at 4 °C until analysis.

Pasteurization was included as an intermediate process control, as preliminary experiments demonstrated that lactic acid bacteria were unable to ferment unpasteurized press cake samples. This group allowed differentiation between thermal effects of pasteurization alone and the biochemical effects attributable to lactic acid fermentation.

For the fermented samples (FER), 500 g of each press cake formulation (H100, H75:O25 and H50:O50) was mixed with 1450 mL of distilled water in a 2 L glass jar. The mixtures were first pasteurized as mentioned above—heated to 80 °C using a double boiler over an induction hot plate. The temperature was maintained for 10 min and controlled using a thermometer. The mixture was agitated every minute using a sterile metal table knife. After 10 min, the mixture was immediately cooled to 40 °C by submerging the closed jar in an ice water bath. Then, 0.375 g of lyophilized commercial starter culture Y883 LYO 50 DCU (*Streptococcus thermophilus* and *Lactobacillus delbrueckii* subsp. *bulgaricus* strains) (Danisco, Copenhagen, Denmark) was added to the mixtures, resulting in an inoculation rate of 25 DCU/L, or 100 DCU/kg.

The fermentation was carried out for 4–5 h in an incubator (BINDER, Tuttlingen, Germany) at 40 °C. Every 30 min, the pH was tested using a SevenCompact S220 pH meter (Mettler Toledo, Columbus, OH, USA). Fermentation was stopped when the pH reached approximately 5.2 by placing the sample at 4 °C.

The initial and final pH as well as the fermentation time for each formulation are indicated below:

H100—initial pH 6.50, final pH 5.22, fermentation time 5 h;

H75:O25—initial pH 6.42, final pH 5.20, fermentation time 4 h;

H50:O50—initial pH 6.35, final pH 5.22, fermentation time 4 h.

Titratable acidity and viable LAB counts were not measured for this study and constitute a limitation of the microbial activity assessment.

The resulting mixtures were spread on a tin tray and oven-dried at 60 °C for 24 h. The dry samples were transferred to plastic bags, vacuum sealed and stored at 4 °C until analysis.

Two batches of each formulation × treatment combination were produced, but due to time and resource constraints, only one representative batch was used for further analyses. This is acknowledged as a limitation of the present study.

### 2.2. Chemical Analysis

#### 2.2.1. Nutritional Value

The raw material composition was determined in accordance with standard methods. Moisture content was estimated by the oven-dry method (ISO 712:2024 [27]). Fat content was determined by acid hydrolysis (AOAC 922.06, 2023 [28]), protein content by the Kjeldahl method (ISO 20483:2013 [29]), total fiber by the enzymatic gravimetric method (AOAC 985.29, 2023 [30]) and ash content by incineration (ISO 2171:2023 [31]). Total carbohydrate content was estimated by a differential method:

$$Total\;carbohydrates\left(\%\right)=100\%-[Moisture\left(\%\right)+Fat\left(\%\right)+Protein\left(\%\right)+Ash\left(\%\right)]$$

Measurements were performed in duplicates. Subsequently, results were normalized on a dry matter basis by excluding moisture content and recalculating the relative proportions of the remaining components.

The nutritional value of the RAW plant-based press cake mixed formulations was estimated by prorating the content of each component in the raw materials. Nutritional composition results are shown in Table 1.

#### 2.2.2. Free and Total Amino Acid Measurement

The amino acid (AA) content in samples was analyzed by ultrafast liquid chromatography (UFLC) with automated o-phthalaldehyde (OPA)/9-fluorenylmethyl chloroformate. Standard solutions of the amino acids alanine (ala), aspartic acid (asp), arginine (arg), cysteine (cys), glycine (gly), valine (val), leucine (leu), isoleucine (ile), threonine (thr), serine (ser), proline (pro), methionine (met), glutamic acid (glu), phenylalanine (phe), lysine (lys), histidine (his), tyrosine (tyr), asparagine (asp) and tryptophan (trp) were analyzed (A9781 Sigma-Aldrich, Schnelldorf, Germany).

Free amino acids were extracted using 0.1 N HCl containing 2% thiodiglycol. An appropriate amount of sample (1–5 g) was homogenized in the extraction solution for 60 min, precipitated with 6% sulfosalicylic acid, and filtered; the pH of the clarified extract was adjusted to 2.20 with NaOH (7.5 M) and diluted to 100 mL with citrate buffer. Prior to analysis, the samples were filtered through 0.22 µm membrane filters.

Free amino acids were separated by UHPLC (YMC-Triart C18, 1.9 µm) on a UFLC system (Shimadzu, Kyoto, Japan) with fluorescence detection and automated OPA/FMOC/MERC derivatization. Mobile phases were solvent A, 20 mmol/L potassium phosphate buffer (pH 6.5), and B, acetonitrile/methanol/water (45/40/15, *v*/*v*/*v*), at 0.5 mL/min and 45 °C. Detection was at Ex/Em 350/450 nm (OPA; primary amino acids) and 266/305 nm (FMOC; proline). A five-level calibration set was used, covering a concentration range of 9.375–150.00 μmol/L for most amino acids and 8.08–75.00 μmol/L for cysteine. The calibration standards (AAS18-10 × 1 mL, Sigma-Aldrich, Germany) were treated identically to the samples, including derivatization. The concentrations of free amino acids in the samples were calculated based on the external calibration curves and expressed in appropriate units considering all dilution factors.

Total amino acid content was determined by acid hydrolysis followed by UFLC with automated o-phthalaldehyde (OPA)/9-fluorenylmethyl chloroformate (FMOC)/Mercaptopropionic Acid (MERC) derivatization. Samples (0.1–0.4 g) were hydrolyzed in 6 M HCl (24 h, 103 °C), neutralized with NaOH (7.5 N) to pH 2.20 below 40 °C, and filtered through 0.45 µm filters prior to injection.

Amino acids were separated by UHPLC (YMC-Triart C18, 1.9 µm) on a UFLC system (Shimadzu, Kyoto, Japan) with fluorescence detection and automated OPA/FMOC/MERC derivatization. Mobile phases were solvent A, 20 mmol/L potassium phosphate buffer (pH 6.5), and B, acetonitrile/methanol/water (45/40/15, *v*/*v*/*v*), at 0.5 mL/min and 45 °C. Detection was RF-20Axs Ex/Em 350/450 nm to Ex/Em 266/305 nm (9.0 min). A five-level calibration set was used, covering a concentration range of 9.375–150.00 μmol/L, except for cysteine covering a concentration range of 8.08–75.00 μmol/L.

Chromatographic analysis was performed strictly adhering to the manufacturer’s standardized guidelines (Shimadzu Corporation, Kyoto, Japan), specifically the pre-validated standard application methodology for high-speed, high-resolution analysis of pre-column derivatized amino acids using the Nexera SIL-30AC autosampler (Shimadzu Corporation, Kyoto, Japan), which provides inherent peak area repeatability through automated constant reaction time control. Quantification was calibrated using fresh external standard curves prepared for each batch, demonstrating high linearity (R^2^ > 0.99). LOD and LOQ values were calculated from the calibration curves as 3.3 and 10 times the standard error of the intercept divided by the slope, respectively. LOD/LOQ calculated results can be found in the Supplementary Materials. A dedicated full validation protocol including replicate injections for precision and matrix spiking for recovery was not performed, as the method follows a pre-validated manufacturer application for this instrumentation. Measurements were performed in duplicates.

#### 2.2.3. Biogenic Amines

Biogenic amines (BAs) were identified and quantified by HPLC. For extraction, 5 g of sample was homogenized with 20 mL of 0.4 mol/L perchloric acid in a 50 mL screw-cap tube, and 250 µL of internal standard (1,7-diaminoheptane, C7H18N2, Sigma-Aldrich, Schnelldorf, Germany) was added from a 1 mg/mL stock solution to achieve a concentration of 1 µg/mL in the injection volume. The mixture was centrifuged at 4000 rpm, and the supernatant was filtered through Whatman No. 1 filter paper; the filtrate was adjusted to 25 mL with 0.4 mol/L perchloric acid.

For dansyl chloride derivatization, 500 µL of extract was alkalinized with 100 µL of 2 mol/L NaOH and buffered with 150 µL saturated NaHCO_3_. Then, 1 mL of dansyl chloride solution (1 mL, 10 mg/mL) was added, mixed thoroughly, and incubated at 40 °C for 45 min. After cooling to room temperature, residual dansyl chloride was quenched with 50 µL of 25% ammonia for 30 min. The volume was adjusted to 5 mL with ammonium acetate/acetonitrile (0.1 mol/L, 1:1, *v*/*v*), and the solution was filtered through a 0.20 µm nylon filter prior to injection.

Chromatographic separation was performed on a Shimadzu Prominence LC20AD system with UV detection at 254 nm (Shimadzu Corporation, Kyoto, Japan), using a Hydrosphere C18 column (5 µm, 12 nm, 150 × 4.6 mm I.D., YMC, Kyoto, Japan). Mobile phase A was 0.1 mol/L ammonium acetate and phase B was acetonitrile. Operating conditions were: flow rate 0.9 mL/min, injection volume 20 µL, column temperature 40 °C, gradient 50–90% B over 19 min, run time 20 min, and post-run 50% B for 8 min.

Six BAs were quantified: putrescine, cadaverine, histamine, tyramine, spermine and spermidine. Standard stock solutions (1 mg/mL) were prepared in 0.1 N HCl and stored at 4 ± 1 °C. Quantification was performed by the external standard method, and standard curves with correlation coefficients were obtained. Measurements were performed in duplicates. Results are expressed in mg·kg^−1^.

#### 2.2.4. Protein Oxidation Measurement

The protein oxidation levels were measured by analyzing the carbonyl content of the samples as a marker of protein oxidation, using the methods and equation described by Soglia et al. (2016) [32]. Briefly, 1 g of each sample was extracted by a 10 mL KCl solution (0.15 M) and homogenized at 15,000 rpm for 1 min. Then, proteins were precipitated using 1 mL trichloroacetic acid (TCA) (10% *w*/*v*) for 15 min. Samples were centrifuged at 5000× *g* for 5 min, and supernatants were discarded carefully, leaving the precipitated protein pellet at the bottom of the tubes. After addition of 400 µL of a sodium dodecyl sulfate (SDS) solution (5% *w*/*v*), the pellets were incubated at boiling point (100 °C) in a water bath for 10 min then cooled to room temperature and stored in the refrigerator (4 °C) overnight. The pellets were treated using 0.8 mL of a 2,4-dinitrophenylhydrazine (DNPH) solution (0.3% *w*/*v* in HCl 3 M) for 1 h. At the same time, blanks were prepared by adding 0.8 mL of HCl 3 M instead. After 1 h of incubation, 400 µL of TCA (40% *w*/*v*) was added to each tube, and the proteins were allowed to precipitate for 15 min. Samples were centrifuged at 5000× *g* for 5 min, and supernatants were discarded gently. Each pellet was washed three times with 1 mL of an ethanol/ethyl acetate mixture (1:1 *v*/*v*) then dried using vac-evaporation for 10 min at 50 °C and 1000 rpm. Pellets were then redissolved in 1.5 mL of a guanidine hydrochloride solution (6 M GuHCl in 20 mM Na_2_HPO_4_ buffer, pH 6.5). Absorbance at 280 nm and 370 nm was used to quantify protein concentration and carbonyl content, respectively, using a Cary 60 UV-VIS spectrophotometer (Agilent Technologies Inc., Santa Clara, CA, USA). The carbonyl content was expressed as nmol/mg of protein and calculated by the following equation:

(1) $$Carbonyl\;content=\frac{A370-A370(blank)}{22,000\times [A280-(A370-A370\left(blank\right))\times 0.43]}\times 10^{6}$$

All chemicals were of analytical grade and obtained from Sigma-Aldrich (JSC Labochema, Lithuania). Measurements were performed in triplicate and averaged.

#### 2.2.5. Protein Solubility (Protein Bonds) Analysis

Analysis of soluble proteins from the raw materials was carried out as described by Chiang et al. (2019) [33]. In 50 mL centrifuge bottles, 0.5 g of dry sample was weighed, and 10 mL of extracting solution was added. The extraction was carried out with agitation for 30 min. The extraction solutions were selected to target bonding interactions as follows:

1. Native state protein (NSP): 0.1 M potassium phosphate buffer, pH 7.5;
2. Hydrogen bonds (HBs): 0.1 M potassium phosphate buffer, pH 7.5, with 8 M urea;
3. Disulfide bonds (DISBs): 0.1 M potassium phosphate buffer, pH 7.5, with 0.05 M dithiothreitol (DTT);
4. Hydrophobic interactions (HYDXs): 0.1 M potassium phosphate buffer, pH 7.5, with 1.5 g/100 mL sodium dodecyl sulfate (SDS);
5. Hydrogen and disulfide bonds (HBs × DISBs): 0.1 M potassium phosphate buffer, pH 7.5, with 8 M urea and 0.05 M DTT;
6. Hydrogen bonds and hydrophobic interactions (HBs × HYDXs): 0.1 M potassium phosphate buffer, pH 7.5, with 8 M urea and 1.5 g/100 mL SDS;
7. Disulfide bonds and hydrophobic interactions (DISBs × HYDXs): 0.1 M potassium phosphate buffer, pH 7.5, with 0.05 M DTT and 1.5 g/100 mL SDS;
8. All bonds and interactions (HBs × DISBs × HYDXs): 0.1 M potassium phosphate buffer, pH 7.5, with 8 M urea, 0.05 M DTT and 1.5 g/100 mL SDS.

The mixture was homogenized using an HG-15D homogenizer (witek Labortechnik GmbH, Wertheim, Germany) at 13,500 rpm for 1 min, followed by agitation for an additional 30 min. Samples were then centrifuged at 4500 rpm for 10 min. The resulting supernatant was filtered through a 0.45 µm pore size nylon syringe filter (25 mm diameter, Agilent, Santa Clara, CA, USA). Soluble protein content was determined using the Bradford protein assay. Absorbance was measured at 595 nm using plastic macro-cuvettes and a Cary UV-60 spectrophotometer (Agilent, Santa Clara, CA, USA). Protein solubility was expressed as g per 100 g of protein, calculated using a bovine serum albumin (BSA) standard curve. Measurements were performed in triplicate and averaged.

#### 2.2.6. SDS-PAGE Analysis

Dry samples were ground using a mortar and a pestle, and 0.5 g was weighed into 50 mL centrifuge tubes. The protein fraction was extracted using 10 mL of Tris buffer 62.5 mM + 2% SDS at pH 8.5. The mixture was homogenized at 1500 rpm for 40 s, then incubated at 90 °C for 60 min. The tubes were centrifuged at 15,000× *g* for 10 min at room temperature. The supernatant was recovered for further protein determination.

The protein content of each sample extraction solution was determined using the Lowry method using the BioRad^®^ DC Protein Assay (Biorad, Hercules, CA, USA). The samples were diluted to a concentration of approximately 1.54 µg/µL. Then, 65 µL of each sample dilution was added to 25 µL of NuPAGE^®^ LDS sample buffer (4×) and 10 µL of Milli-Q water (non-reducing conditions) or NuPAGE^®^ Sample Reducing Agent (reducing conditions). The mixtures were heat-treated at 70 °C for 10 min, and 10 µL was loaded onto NuPAGE^®^ 12% Bis-Tris Gels (Thermo Scientific, Waltham, MA, USA). As a marker for protein molecular weight calibration, a PageRuler Broad Range Unstained Protein Ladder (Thermo Scientific, Waltham, MA, USA) 5–250 kDa was used. The gels were settled in an XCell SureLock Mini-Cell (Thermo Scientific, Waltham, MA, USA) electrophoresis chamber, and the electrophoresis was carried out in 3-(N-morpholino)propanesulfonic acid (MOPS) 1× running buffer at 200 V for 1 h. The resulting gels were heat-stained by placing them in a solution consisting of 40% ethanol, 10% acetic acid and 1% Coomassie blue and microwaving them at low heat for 1 min. The gels were destained by heat treatment for 1 min at low heat in a 10% ethanol, 7.5% acetic acid solution. For better resolution, the gels were left in distilled water in a closed container for 48 h before being photographed for analysis.

For each SDS-PAGE gel, a lane of dry extruded pure hemp sample was included as an additional control of heat treatment (noted as H100 EXT). Three gels were run under the same conditions, and the gel images presented in the article correspond to a representative run.

#### 2.2.7. Phytic Acid Content Determination

The phytic acid content was determined with a Phytic Acid Assay Kit (Megazyme, Bray, Ireland) following the manufacturer’s instructions. The extraction step was carried out following the considerations described in the section “Modified sample extraction for samples with A655 above STD 4”. Measurements were performed in triplicate and averaged.

### 2.3. Water-Holding Capacity (WHC) and Oil-Holding Capacity (OHC)

WHC and OHC were determined using the method described by Sadh et al. [34] with some modifications. Slurries were prepared in previously weighed 15 mL centrifuge tubes by adding 0.8 g of sample and 12 mL of distilled water and shaking the mixture manually for 1 min. Then, the tubes were centrifuged for 10 min at 3000× *g*. After centrifugation, the supernatant was discarded, and the tubes were weighed again. The WHC or OHC was expressed as grams of water or oil, respectively, bound per gram of sample. Measurements were performed at least in triplicate. For samples with very high result variability, WHC and OHC determination was performed in several replicates, up to 12 times.

### 2.4. Particle Size Distribution Analysis

The particle size distributions of the raw materials and formulations were measured using a Malvern Mastersizer 3000 (Malvern Instruments Ltd., Worcestershire, UK) equipped with an Aerocell S accessory for dry measurements. Measurement settings were as follows: non-spherical particles, refractive index 1.46, obscuration range 5–20%, and data acquisition time of 30 s. All samples were measured in triplicate and averaged. The collected data were Dv10, Dv50 and Dv90.

### 2.5. Statistical Analysis

Data analysis was done using R (4.5.2) and RStudio (2026.01.2). Given that only one independently prepared batch per formulation × treatment combination was available for analysis, with measurements performed in duplicate or triplicate as analytical replicates within this single batch, the dataset does not meet the requirements for independent biological replication necessary for standard inferential statistical testing. Accordingly, all analyses should be interpreted as exploratory and descriptive rather than confirmatory. ANOVA-derived *p*-values are reported for descriptive purposes only and should not be interpreted as the basis for inferential conclusions; generalized eta-squared (ges) is reported as the primary descriptor of the observed effect magnitude within this dataset.

For protein oxidation, protein solubility, OHC and phytic acid determination, a two-way analysis of variance (ANOVA) with matrix formulation and treatment as fixed factors was applied, using Tukey’s honestly significant difference (HSD) test for post hoc pairwise comparisons to characterize the pattern of observed differences. For WHC, the Welch ANOVA was used instead, due to non-homogeneous variances between groups, and the Games–Howell test was used for pairwise comparisons.

For biogenic amines and free and total amino acid content, two-factor ANOVA with formulation and treatment as fixed factors was applied, with generalized eta-squared (ges) reported as the primary measure of observed effect magnitude. Where normality assumptions were not met, the Scheirer–Ray–Hare non-parametric test was used as an alternative. Post hoc pairwise comparisons were performed using Tukey’s honestly significant difference (HSD) test or Dunn’s test with Bonferroni correction for multiple comparisons. Two-dimensional principal component analysis (PCA) was additionally applied as a complementary visualization of multivariate patterns, using the FactoMineR and factoextra packages. All findings should be considered preliminary and subject to confirmation in studies with independently replicated batches.

## 3. Results and Discussion

### 3.1. Interplay Between Proteolysis, Amino Acid Release, and Biogenic Amine Formation

#### 3.1.1. Total Amino Acids

The total amino acid content of the press cake samples was evaluated for nineteen amino acids (aspartic acid, asparagine, glutamic acid, serine, glycine, histidine, threonine, alanine, arginine, proline, cysteine, tyrosine, valine, methionine, lysine, isoleucine, leucine, phenylalanine and tryptophan) across three matrix formulations (H100, H75:O25 and H50:O50) and three treatments (RAW, PAS, FER). Due to conventional acid hydrolysis conditions, asparagine and glutamine were quantitatively deamidated and are reported as combined fractions: asx (asp + asn) and glx (glu + gln). Tryptophan was completely degraded during acid hydrolysis and was excluded from the profile. Cysteine and methionine were quantified directly from the hydrolysate without pre-oxidation; therefore, their reported values represent conservative underestimations due to partial oxidative degradation. For amino acids where normality assumptions were not met (ser, his, thr, cys), the Scheirer–Ray–Hare test was used in place of two-factor ANOVA.

For the sum of total amino acids, two-factor ANOVA indicated large observed effects of formulation (ges = 0.979), treatment (ges = 0.830), and their interaction (ges = 0.900) (see Table 2), suggesting that the observed differences associated with matrix composition and processing were not independent of each other within this dataset.

At the individual amino acid level, large observed effects of both formulation and treatment were noted for the majority of amino acids. Formulation was associated with the largest observed effect sizes for asx, glx, gly, ala, arg, tyr, val, lys, ile, leu, and phe (ges ranging from 0.845 to 0.978), reflecting the strong association between the HPC:OPC ratio and overall protein composition within the dataset. Higher HPC content was consistently associated with higher total amino acid levels. In contrast, met and pro showed small observed formulation effect sizes (ges = 0.059 and 0.423 respectively), suggesting these amino acids are less sensitive to matrix composition under the studied conditions.

Treatment was associated with large observed effects for a partially overlapping set of amino acids, including asx, glx, gly, his, ala, arg, pro, cys, tyr, met, lys, ile, and leu, with notably large effect sizes for ala (ges = 0.980), glx (ges = 0.961), and lys (ges = 0.918). Val and phe showed very small treatment effect sizes (ges = 0.107 and 0.010, respectively), consistent with a relatively stable content across RAW, PAS, and FER conditions regardless of formulation.

Notable interaction effect sizes were observed for glx, gly, ala, arg, pro, tyr, val, lys, ile, leu, and phe, suggesting that the observed effect of processing may be formulation-dependent for these amino acids. Notably, val and phe showed large interaction effect sizes (ges = 0.843 and 0.923, respectively) despite small main treatment effects, suggesting that treatment-related changes in these amino acids may have been present but opposing across formulations, partly canceling at the overall level.

Post hoc comparisons (see Appendix A, Table A1) suggested that, within this dataset, pasteurized samples showed a distinct profile relative to RAW and FER samples, which were more similar to each other, particularly in combined formulations. Among individual amino acids, proline showed a notable pattern: pasteurization was not associated with a meaningful observed change, whereas fermentation was associated with increased content in both combined formulations. Lysine showed an apparent downward trend across processing steps, with lower observed values after pasteurization and further after fermentation. Ser, thr, cys, and met showed small observed pairwise differences between treatment stages within the same formulation in post hoc comparisons, suggesting that processing was not associated with meaningful separation between RAW, PAS, and FER samples for these amino acids.

**Figure 1:**
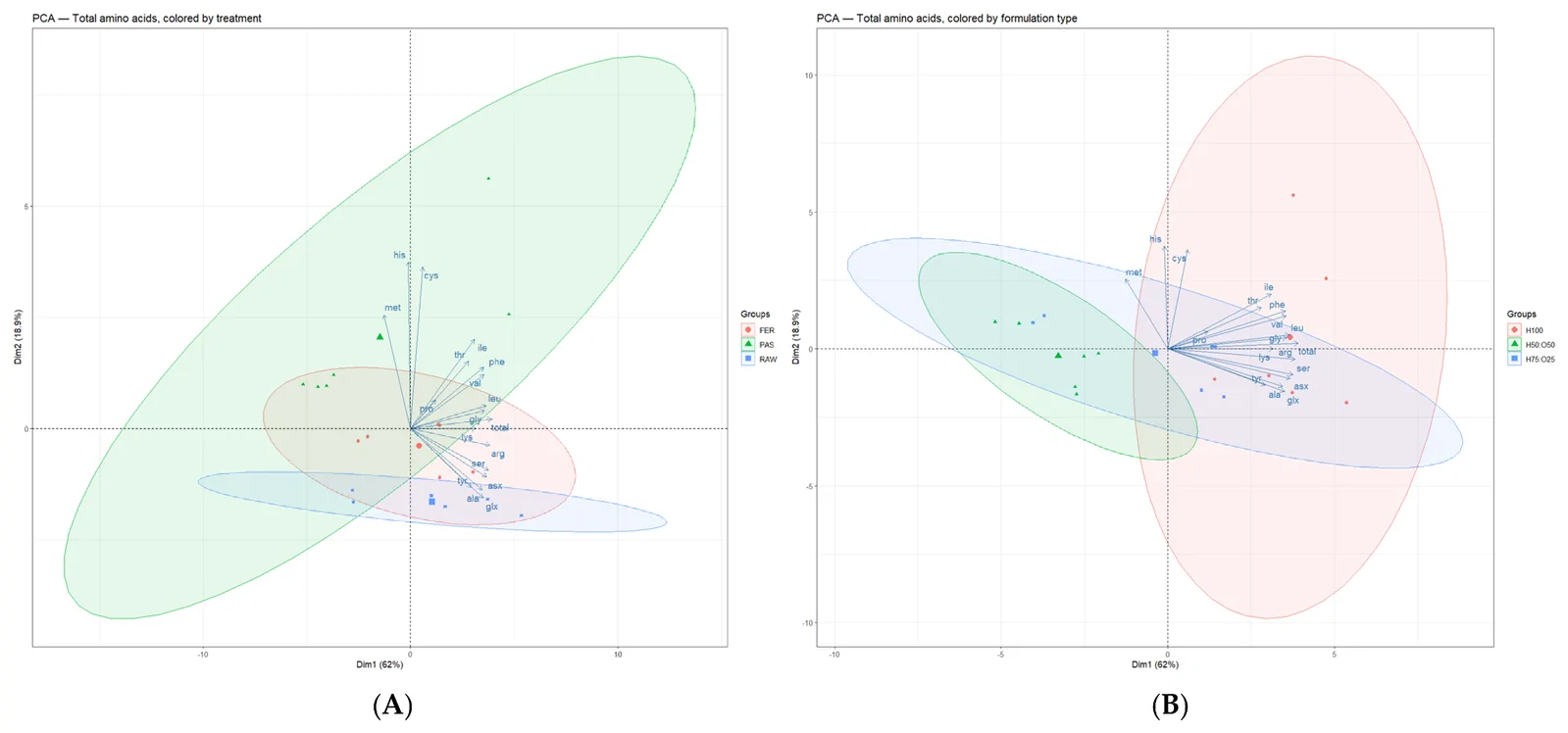
Principal component analysis of total amino acid content. Biplots of component 1 (PC1, 59.9%) versus component 2 (PC2, 20.0%), illustrating the relationships between the measured variables. (**A**) Biplot with samples clustered by treatment. (**B**) Biplot with samples clustered by formulation.

Overall, formulation was associated with the largest observed differences in total amino acid composition within this dataset, with higher HPC content consistently associated with higher amino acid levels. Treatment-associated differences were amino-acid-dependent rather than uniform. PCA (Figure 1, Table A7), explaining 79.8% of the total variance, provided a complementary visualization of these patterns, with hemp-heavy formulations consistently separated along PC1, reflecting differences in overall protein content rather than shifts in specific amino acid classes.

#### 3.1.2. Free Amino Acids

The free amino acid content was evaluated for nineteen amino acids across the same formulations and treatments. For amino acids where normality assumptions were not met (asp, glu, ser), the Scheirer–Ray–Hare test was used in place of two-factor ANOVA.

For the sum of free amino acids, the two-factor ANOVA (see Table 3) indicated a dominant observed effect of treatment (ges = 0.964), a considerably smaller observed effect of formulation (ges = 0.588), and a notable interaction between both factors (ges = 0.914), suggesting that the observed effect of processing on free amino acid release was formulation-dependent within this dataset.

At the individual amino acid level, the majority showed large observed effects of treatment, formulation, and their interaction, with ges values frequently exceeding 0.90. The largest treatment-associated effects were observed for leu (ges = 0.992), met (ges = 0.985), ala (ges = 0.982), arg (ges = 0.981), and thr (ges = 0.976), suggesting that these amino acids were the most responsive to processing conditions within this dataset. Formulation-associated effects were also large for most amino acids, with the exception of asp, glu, ser, tyr, and trp, suggesting their free amino acid content was primarily associated with treatment rather than matrix composition. Trp showed small observed effects for all factors, and tyr showed a large treatment effect size (ges = 0.764) with negligible formulation and interaction effect sizes.

Large interaction effect sizes between formulation and treatment were observed for the majority of amino acids, with the largest values for arg (ges = 0.992), leu (ges = 0.988), met (ges = 0.981), ala (ges = 0.973), and thr (ges = 0.955), consistent with strongly divergent observed responses to processing depending on matrix composition.

Post hoc comparisons (see Appendix A, Table A2) suggested that the direction of observed treatment effects differed between pure and combined formulations within this dataset. In H100, both pasteurization and fermentation were associated with lower overall free amino acid content relative to RAW, with the largest observed decreases for asn, ala, arg, and tyr. In combined formulations, pasteurization was similarly associated with reduced free amino acid content, while fermentation was associated with subsequent increases to levels similar to or even above RAW, with particularly pronounced changes observed in asn, ala, pro, val, lys and leu.

Among fermented samples, H100F showed the lowest free amino acid content, in contrast to H75:O25F and H50:O50F. This divergent response may reflect the limited carbohydrate availability in the pure hemp matrix, as discussed further in Section 3.1.4. Contrarily, H75:O25 showed a fermentation response comparable to H50:O50 in terms of overall free amino acid release.

**Figure 2:**
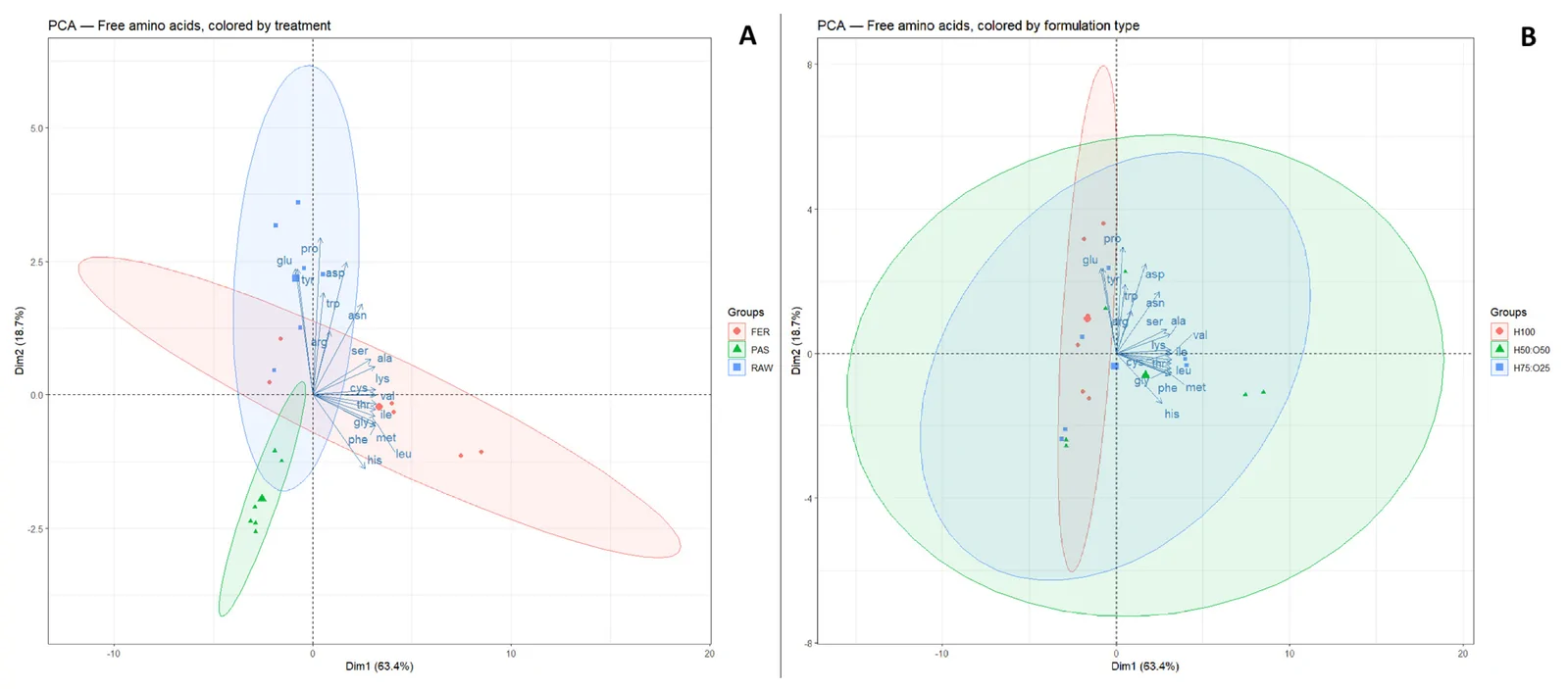
Principal component analysis of free amino acid content. Biplots of component 1 (PC1, 63.4%) versus component 2 (PC2, 18.7%), illustrating the relationships between the measured variables. (**A**) Biplot with samples clustered by treatment. (**B**) Biplot with samples clustered by formulation.

The PCA of free amino acids (Figure 2, Table A8), explaining 82.1% of total variance, provided a complementary overview of these patterns, with treatment dominating variation along PC1 and formulation having a more modest influence on amino acid composition along PC2.

#### 3.1.3. Biogenic Amines

Biogenic amine content was evaluated for six amines (histamine, cadaverine, putrescine, tyramine, spermine, and spermidine) across all formulations and treatments. Histamine and cadaverine were excluded from statistical analysis due to near-zero concentrations across all samples. All remaining amines met normality assumptions and were analyzed by two-factor ANOVA.

Two-factor ANOVA (see Table 4) showed very large observable effect sizes of formulation, treatment, and their interaction for all four amines and for total BA content (all ges ≥ 0.950). These results suggest that the observed effects of matrix composition and processing on the biogenic amine profile were not independent of each other within this dataset. Spermidine showed the largest observable effects across all three terms (formulation ges = 1.000; treatment ges = 1.000; interaction ges = 1.000), suggesting strong associations between content and these two factors. Similarly large effects were observed for spermine (formulation ges = 0.980; treatment ges = 0.999; interaction ges = 0.991) and putrescine (formulation ges = 0.996; treatment ges = 0.973; interaction ges = 0.980). Tyramine showed comparatively smaller, though still large, effect sizes (formulation ges = 0.965; treatment ges = 0.994; interaction ges = 0.982).

Post hoc comparisons (see Appendix A, Table A3) showed that H100 had the highest total BA content among all formulations, driven primarily by elevated spermine or spermidine levels. Spermine and spermidine showed opposing trends across treatments in all formulations: pasteurization was associated with higher spermine levels and lower spermidine levels relative to RAW, while fermentation was associated with an opposite pattern, showing lower spermine and higher spermidine values, in some cases to levels equal to or exceeding RAW.

Putrescine and tyramine also seemed to show divergent observable responses to processing. In combined formulations, processing steps were associated with a progressively higher content of putrescine, whereas in H100 pasteurization was associated with a lower content of this amine and subsequent fermentation was associated with a moderate putrescine recovery. Tyramine showed an inverse pattern across all formulations: pasteurization was associated with a higher content relative to RAW, while fermentation was associated with a lower content, closer to baseline levels.

**Figure 3:**
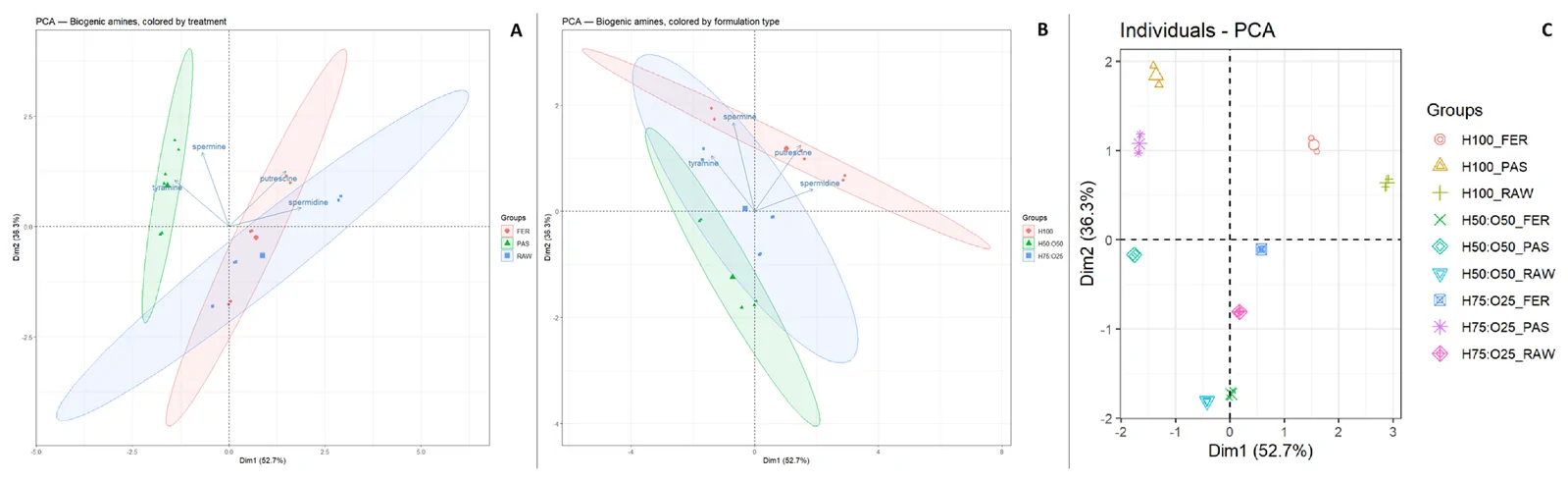
Principal component analysis (PCA) of four individual biogenic amine contents. Biplots of component 1 (PC1, 52.7%) versus component 2 (PC2, 36.3%) illustrating the relationships between the measured variables. (**A**) Biplot with samples clustered by treatment. (**B**) Biplot with samples clustered by formulation. (**C**) Individual plot.

The PCA (Figure 3, Table A9) is presented as a complementary visualization of these patterns, consistent with the large observable effects of treatment and formulation, and illustrates the largely orthogonal nature of their contributions to overall amine profile variation.

#### 3.1.4. Integrated Discussion

The combined analysis of total amino acids, free amino acids, and BAs provides a comprehensive view of nitrogen transformations occurring during processing of hemp–oat formulations. These datasets represent successive stages of protein metabolism, from initial composition to proteolytic breakdown and subsequent conversion to biogenic amines.

For total amino acids, formulation was associated with bigger compositional differences, with effect sizes consistently among the largest observed across the entire dataset (ges up to 0.979 for the sum). This is consistent with the distinct protein profiles of hemp and oat press cakes, with a higher HPC content associated with a greater overall amino acid content. Treatment effects on total amino acids were present but amino-acid-dependent, and post hoc comparisons indicated that pasteurized samples were most distinct from RAW and FER, which remained relatively similar to each other. This suggests that thermal treatment during pasteurization was more strongly associated with differences in total amino acid composition than fermentation, possibly through protein denaturation and aggregation effects on extractability rather than true amino acid loss or gain.

In contrast, for free amino acids, treatment showed the strongest association (ges = 0.964 for the sum), with formulation playing a secondary but notable role (ges = 0.588). The large observable interaction effects for most individual amino acids (ges frequently exceeding 0.95) suggest that the response to processing was strongly matrix-dependent within this dataset. This divergence between pure and combined formulations is one of the most notable observed patterns in this dataset. In H100, both pasteurization and fermentation were associated with a lower free amino acid content relative to RAW, whereas in combined formulations, the opposite was true and fermentation was associated with free amino acid content above RAW levels. The amino acids most responsible for this divergence (asn, ala, arg, pro, and lys) are among those showing the largest interaction effect sizes, suggesting that their behavior under fermentation conditions may differ depending on matrix composition.

The lower free amino acid content observed in H100F relative to all other fermented samples is consistent with the hypothesis that, in the absence of sufficient carbohydrate substrate, amino acids may serve as alternative energy sources during fermentation, as has been reported for lactic acid bacteria in carbohydrate-limited media [35,36]. The limited carbohydrate content of HPC and the limited observed fiber reduction after fermentation in this matrix are consistent with this interpretation, though confirmation through carbohydrate and metabolite profiling, as well as microbiological characterization, is required. The fact that even H75:O25 showed a fermentation response comparable to H50:O50 in terms of net free amino acid is consistent with the possibility that even a relatively modest oat press cake inclusion may shift fermentation dynamics toward proteolytic amino acid liberation rather than amino acid consumption within the study conditions.

Despite the fermentation-associated increases in free amino acid content in combined formulations, biogenic amine formation did not seem to mirror this trend. The ANOVA for all four amines and total BA content showed near-maximal observed effect sizes for formulation, treatment, and their interaction (ges approaching or reaching 1.000 for spermidine and total BA), suggesting that amine accumulation was strongly associated with both factors in this dataset, with neither acting independently.

The opposing patterns of spermine and spermidine across treatments, where pasteurization was associated with increased spermine and decreased spermidine, and fermentation was associated with the reverse pattern, is consistent with known interconversion relationships between polyamines, where these compounds can be interconverted through oxidative and back-conversion pathways [37]. However, the specific enzymatic activities driving these shifts were not measured in this study, and the microbial contributions to these conversions cannot be confirmed without microbiological characterization. The divergent behavior of putrescine between H100 and combined formulations further suggests that matrix composition shapes the conditions under which specific conversion pathways operate, though whether this reflects differences in precursor availability, enzymatic activity, or microbial community composition remains to be established [38,39,40].

Altogether, these preliminary observations are consistent with protein degradation and BA formation being governed by different factor hierarchies and not being linearly coupled processes. Total amino acid composition is primarily associated with matrix formulation. Free amino acid release is primarily associated with treatment but also with formulation through interaction effects. Biogenic amine accumulation showed near-complete association with both factors within this dataset, with amine-specific directionality suggesting selective conversion rather than generalized substrate-driven accumulation. The mechanistic basis of these conversions, particularly the enzymatic and microbial drivers, requires targeted metabolomic and microbiological approaches beyond the scope of this study and represents an important direction for future work given the nutritional and safety implications of both free amino acid availability and biogenic amine accumulation in fermented plant-based matrices.

### 3.2. Protein Oxidation

**Figure 4:**
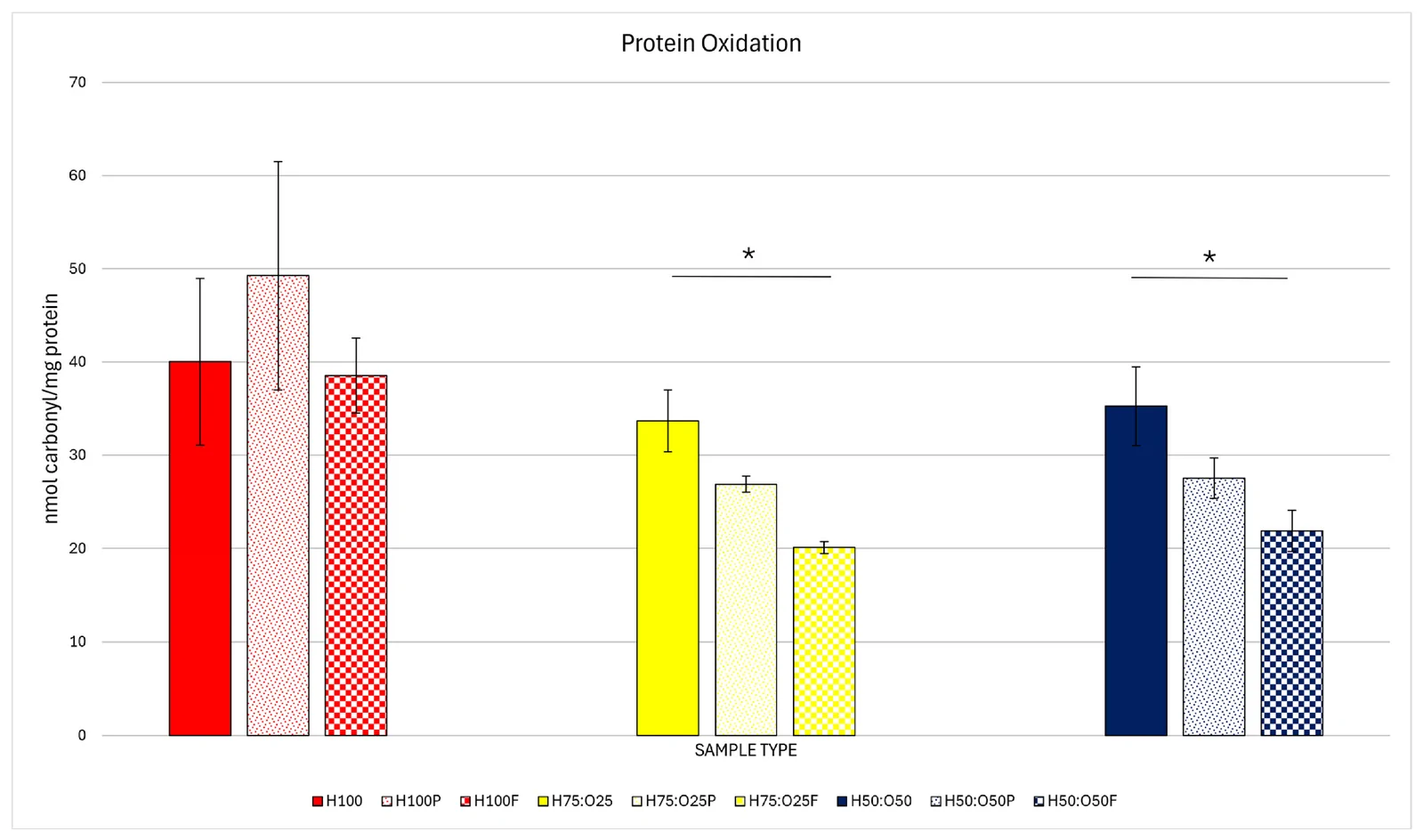
Protein oxidation of plant-based combined matrices calculated as carbonyl content by DNPH method (H100: hemp press cake, H75:O25: formulation 75% hemp press cake and 25% oat press cake, H50:O50: formulation 50% hemp press cake and 50% oat press cake, P: pasteurized, F: fermented). The error bars represent the standard deviation (SD). The horizontal bars with a (*) symbol above indicate notable observed pairwise differences between samples of the same formulations.

Protein oxidation, assessed through carbonyl quantification, was affected by both matrix formulation and processing treatment (Figure 4, Table A10). Formulation showed the largest observed effect (ges = 0.705) compared to treatment (ges = 0.444). The interaction observed effect was considerably smaller (ges = 0.338), and simple effects analysis revealed that treatment was associated with slightly higher observable effects in mixed formulations (H75:O25: ges = 0.326; H50:O50: ges = 0.321) than in pure hemp (H100: ges = 0.261). Additionally, formulation differences between samples were only associated with protein oxidation changes after processing, the carbonyl content trend being RAW > PAS > FER for mixed formulations, whereas RAW samples showed a comparably smaller simple effect of formulation (ges = 0.103, compared to 0.63 and 0.521). However, post hoc comparisons (Table A4) showed small differences between adjacent processing steps (RAW vs. PAS and PAS vs. FER), with only the RAW vs. FER comparison being notable in combined formulations, suggesting a gradual rather than stepwise reduction in carbonyl content. The carbonyl content was highest in H100P (49.3 nmol carbonyl/mg protein) and lowest in H75:O25F (20.1 nmol carbonyl/mg protein) (Figure 4), suggesting a reduction in protein carbonylation under fermentation conditions in combined matrices, though the specific mechanisms driving this reduction were not characterized.

Protein oxidation reflects changes in protein quality within the matrices studied. Food processing, particularly heat and mechanical treatment, can increase oxidation and alter protein structure. During thermochemical processing, reactive species react with amino acid side chains, leading to the formation of protein carbonyls, disulfide bonds, dityrosine and protein radicals [41]. While some oxidation is unavoidable, extensive oxidation can reduce protein solubility and functionality, thereby reducing nutritional value. Fermentation has been previously reported to reduce oxidation markers in plant-based matrices [42].

In the present study, H100 samples presented the highest levels of protein oxidation, and neither pasteurization nor fermentation processes were associated with notable changes in this parameter. This suggests that the processing treatments were insufficient to reduce the protein oxidation already present in the raw HPC. Hemp seeds are typically steam-pasteurized before pressing, exposing them to high temperature and humidity. Although most oil is removed by pressing, the resulting HPC still contains a considerable fat fraction (>10%), which is prone to lipid oxidation and can promote protein oxidation. The lipid fraction of hempseed is rich in polyunsaturated fatty acids, which are highly susceptible to oxidation under the storage conditions [43]. In H100, where the relative amount of hemp-derived lipids is highest, the lipid-driven protein oxidation may be more pronounced, which may explain the elevated carbonyl levels in the RAW sample. The limited observed effect of processing may be associated with non-enzymatic lipid autooxidation pathways, which proceed via radical chain reactions and are less affected by pasteurization or fermentation [41]. Additionally, the oven drying applied to all samples after pasteurization and fermentation (60 °C, 24 h) may have contributed to further protein oxidation through thermally induced carbonyl formation and cannot be fully decoupled from the effects of the processing treatments themselves.

In contrast, the fermentation of combined formulations (H75:O25 and H50:O50) showed a gradual reduction in carbonyl content from RAW to FER. The incorporation of OPC likely dilutes the hemp-derived lipid fraction, reducing lipid peroxidation and subsequent protein oxidation. In RAW mixed formulations, oat-derived antioxidants such as vitamin E, phenolic compounds, and avenanthramides [44] may have a modest effect to lower oxidation levels compared to H100. Pasteurization may further reduce oxidation through heat-induced inactivation of oxidative enzymes such as lipoxygenase. During fermentation, the release of bound phenolics and the formation of low-molecular-weight peptides with antioxidant activity [45,46] may contribute to the additional reduction in carbonyl content. Overall, matrix composition and fermentation have shown associations with oxidative stability, pointing to mixed press cake systems as a possible strategy to enhance protein quality in fermented plant-based matrices. It should be noted, however, that the drying step applied after processing may have contributed to carbonyl accumulation across all processed samples, and future studies should consider alternative sample preservation methods to isolate the contribution of drying from that of fermentation and pasteurization.

### 3.3. Protein Solubility (Protein Bonds)

**Figure 5:**
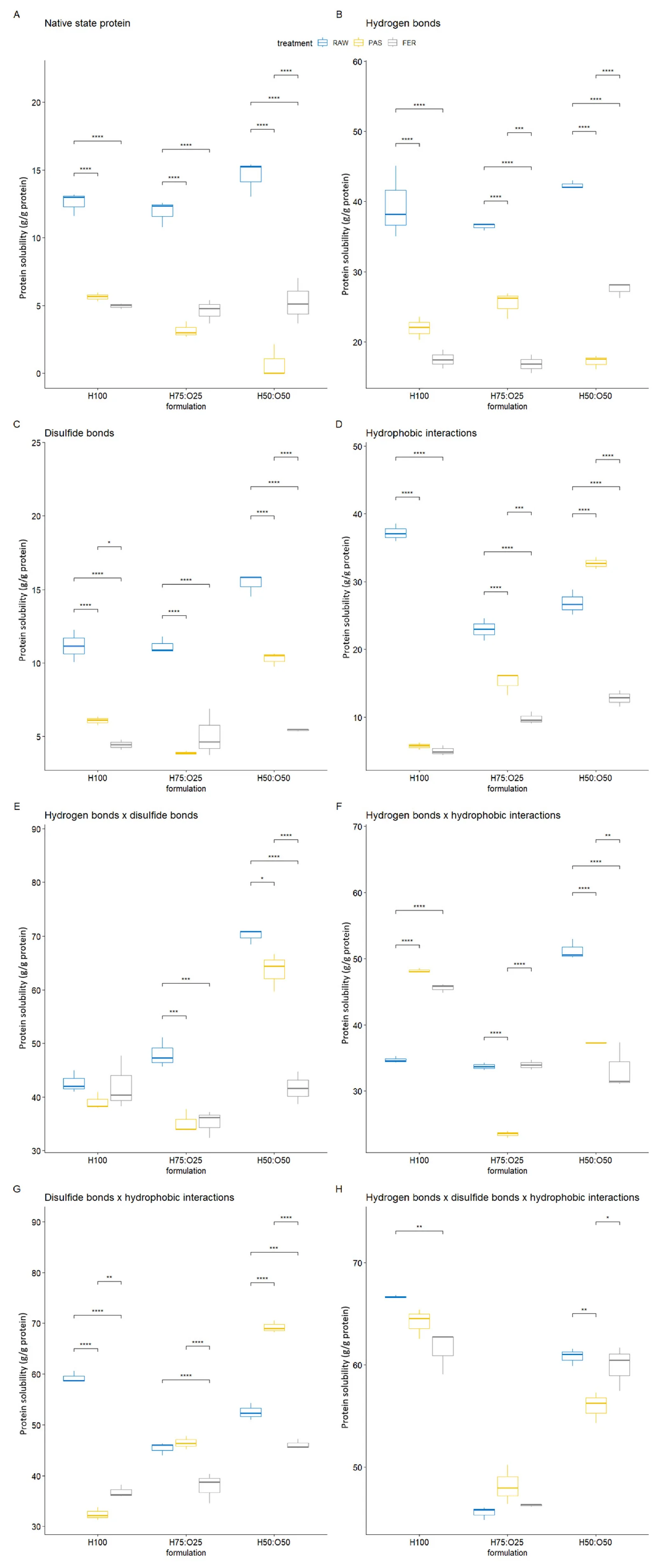
Protein solubility of combined hemp and oat press cake formulations (H100, H75:O25, H50:O50) induced by different extracting solutions dissolving specific chemical bonds (H100: hemp press cake, H75:O25: formulation 75% hemp press cake and 25% oat press cake, H50:O50: formulation 50% hemp press cake and 50% oat press cake, P: pasteurized, F: fermented). (**A**) Native state protein; (**B**) Hydrogen bonds; (**C**) Disulfide bonds; (**D**) Hydrophobic interactions; (**E**) Hydrogen bonds x disulfide bonds; (**F**) Hydrogen bonds x hydrophobic interactions; (**G**) Disulfide bonds x hydrophobic interactions; (**H**) Hydrogen bonds x disulfide bonds x hydrophobic interactions. Asterisks indicate notable pairwise differences based on Tukey-adjusted estimated marginal means, reported for descriptive purposes only (*p* ≤ 0.05 (*), ≤0.01 (**), ≤0.001 (***), ≤0.0001 (****)).

The solubility of proteins extracted under bond-specific conditions (Figure 5, Table A11) suggested substantial remodeling of protein interaction networks as a result of both formulation and treatment.

Across almost all solutions, the treatment effect was associated with very large observed effect sizes (ges often > 0.9). This hints at the strong association of processing with protein structure and solubility. On the other hand, the formulation effect seemed comparatively weak in native conditions (ges 0.271) (Figure 5A, native state protein) but became stronger in disruptive solvents (including single-, double- and triple-bond targeting solvents), suggesting intrinsic protein composition differences that only become visible after structural disruption. The interaction between both factors, formulation and treatment, was often large (ges up to 0.97), which may suggest that processing affects hemp and oat proteins differently.

#### 3.3.1. Native State Protein Solubility

For the phosphate buffer solution (Figure 5A), the formulation observed effect was small (ges = 0.271) compared to the observed treatment effect (ges = 0.965) and the two-factor interactions (ges = 0.697). This suggests that in native conditions, where proteins are mostly aggregated and non-denatured, solubility is strongly associated with treatment rather than matrix composition within this dataset. Post hoc comparisons (Table A5) showed that, across all formulations, RAW samples differed from both PAS and FER, while PAS and FER notably did not differ from each other, suggesting that the main solubility loss occurs upon initial processing rather than progressively across treatment steps. This also reflects the action of both processing steps. Pasteurization may affect protein solubility by playing on the aggregation/denaturation balance of the proteins [47,48], whereas fermentation may induce partial unfolding and hydrolysis by action of microbial agents [49,50], though these mechanisms were not directly characterized in this study. Notably, the calculation of principal effects showed that for the FER samples, the formulation effect was small, suggesting that processing homogenized protein structure across formulations. The pH variation induced by the fermentation process is also worth mentioning. Although fermentation decreased pH toward the isoelectric region (around 5.0 [51,52]), the observed increase in solubility may instead reflect structural rearrangements and partial hydrolysis, which override classical aggregation effects expected at the isoelectric point. As HPC contributes significantly more than OPC to the total protein content of the mixed formulations, the homogenization of protein solubility is also explained. Finally, the potential effect of the drying process on protein aggregation cannot be overlooked and might further contribute to the lower protein solubility values associated with PAS and FER samples.

#### 3.3.2. Hydrogen Bonds

In the urea-containing solution (Figure 5B), disruption of hydrogen bonds was associated with increased solubility overall. Treatment showed a bigger observed effect size (ges = 0.963), whereas formulation was associated with a smaller observable effect (ges = 0.367). The interaction effect between the two factors was also notable within the dataset (ges = 0.807). Across all formulations, post hoc comparisons showed that RAW samples had higher protein solubility than both PAS and FER, with H75:O25 showing a further decrease from PAS to FER. While some formulation-associated effects were observed, suggesting some compositional contribution to hydrogen-bond-related solubility, treatment showed considerably stronger observed effects, suggesting hydrogen bonds play an important role in maintaining protein aggregation and native structure independent of matrix composition.

#### 3.3.3. Disulfide Bonds

Disruption of disulfide bonds using DTT (Figure 5C) showed that treatment was associated with observable effects (ges = 0.965) larger than the formulation effect (ges = 0.873), with a notable interaction between the two (ges = 0.744). In H100 and H50:O50, post hoc comparisons showed stepwise decreasing trends across RAW, PAS, and FER, suggesting that disulfide-bond-related solubility declined progressively with processing. In H75:O25, PAS and FER showed comparable solubility; however, the downward trend from unprocessed to processed is still evident. Notably, solubility values under this solvent condition were close to those observed under native conditions (3.3.1), suggesting limited contribution of disulfide bonds to the overall protein interaction network in these matrices. While formulation observed effects were notable, likely reflecting differences in cysteine content between hemp and oat proteins, treatment-associated observed effects on disulfide-bond-related solubility were the largest, suggesting that processing conditions, rather than matrix composition, primarily govern this aspect of protein structure.

#### 3.3.4. Hydrophobic Interactions

In the SDS-containing solution (Figure 5D), disruption of hydrophobic interactions was associated with notable increases in solubility along with observed effects of both formulation and treatment, with treatment having the largest observed effect (ges = 0.984). Nonetheless, formulation showed a markedly larger observable effect than for the other single-bond conditions (ges = 0.933), alongside a strong interaction effect (ges = 0.976). Post hoc comparisons showed solubility decreases from RAW to FER across all formulations. The difference between PAS and FER was not always evident but showed a consistent downward pattern. However, only H50:O50 showed a notable increase in solubility in PAS compared to RAW. The formulation-associated effect size suggests that hydrophobic interactions represent one of the primary stabilizing forces in the system in which compositional differences between hemp and oat proteins are most clearly expressed among the single-bond conditions, even though processing remains the principal determinant of overall solubility.

#### 3.3.5. Hydrogen Bonds × Disulfide Bonds

When hydrogen and disulfide bonds were disrupted together (Figure 5E), formulation showed a notable observed effect size (ges = 0.930), exceeding the observed effect associated with treatment for the first time among the conditions tested (ges = 0.855), with a strong interaction between the two (ges = 0.844). This marks a shift from the single-bond conditions, where treatment was associated with the strongest effect, suggesting that as multiple non-covalent and covalent interactions are disrupted simultaneously, intrinsic compositional differences between hemp and oat proteins become increasingly important. Post hoc comparisons showed minimal differences between RAW, PAS, and FER within H100, consistent with the smaller observed treatment-associated effect for this formulation. In contrast, mixed formulations showed notable treatment-associated observed effects, with H50:O50 showing observable differences across all three treatment stages. Hemp proteins therefore seem to be more resistant to combined disruption, which may suggest a stronger intrinsic structure, highly stabilized by hydrophobic interactions.

#### 3.3.6. Hydrogen Bonds × Hydrophobic Interactions

When hydrogen bonds and hydrophobic interactions were disrupted simultaneously (Figure 5F), formulation showed a notably stronger observed effect than treatment (ges = 0.961 and ges = 0.645 respectively), with a strong interaction between the two (ges = 0.968). This suggests that once the main non-covalent interactions, two of the primary stabilizing forces identified in the single-bond analyses, are removed together, intrinsic differences between hemp and oat proteins are more strongly associated with the solubility response than processing conditions. Post hoc comparisons showed notable treatment effects within most formulations, although H100 and H75:O25 showed no observable differences between PAS and FER and between RAW and FER, respectively. Solubility was also relatively low in this system compared to other double-bond targeting solvents, suggesting that disulfide bonds still contribute to structural stability once hydrogen bonds and hydrophobic interactions are disrupted.

#### 3.3.7. Disulfide Bonds × Hydrophobic Interactions

When disulfide bonds and hydrophobic interactions were disrupted together (Figure 5G), both formulation (ges = 0.959) and treatment (ges = 0.944) showed strong observed effects, with formulation only marginally exceeding treatment, alongside a strong interaction between the factors (ges = 0.972). Unlike the other dual-disruption conditions, treatment retained a comparably large influence here, suggesting that the combined removal of disulfide bonds and hydrophobic interactions leaves protein structure responsive to both processing and composition to a similar degree. Post hoc comparisons showed observable stepwise differences across RAW, PAS, and FER within most formulations, suggesting that both covalent and non-covalent interactions are important for maintaining protein structure and that removing both may reveal differences between formulations and processing effects.

#### 3.3.8. All Bonds and Interactions

The combination of all solvents was used to estimate protein solubility when all bonds and their interactions are broken (Figure 5H). Formulation showed the greatest observed major effect (ges = 0.975), while the treatment effect became considerably minor (ges = 0.317), with a moderate interaction between the two (ges = 0.664). Post hoc comparisons showed a limited and formulation-associated treatment response under this condition, with H100 showing only a notable RAW-FER difference and H50:O50 showing a marginal PAS-FER difference. This suggests that when all interactions are broken, protein solubility is mainly associated with composition differences between the samples. As expected, the average solubility for this solution was the highest of all solutions, suggesting maximum disruption of all protein bonds.

#### 3.3.9. Integrated Discussion

The two-factor ANOVA of protein solubility across different solvent systems showed that both treatment and formulation influenced protein structural organization, with strong interaction effects observed in all cases. Under native conditions, protein solubility was primarily associated with treatment, suggesting that processing plays an important role in determining protein aggregation and conformational state. Post hoc comparisons additionally suggest that the main solubility loss occurred upon initial processing (RAW to PAS/FER) rather than progressively across treatment stages. Processing conditions, such as temperature and pH, may explain the most observable effects in this case, as their influence on protein aggregation and solubility has been largely documented. The drying post-processing step may also play a role in protein aggregation and therefore on solubility. However, as progressively stronger solvents were applied to disrupt specific interactions, the effect of formulation became increasingly pronounced.

Comparing single-bond disrupting solutions (solutions 2–4), treatment-associated observable effects were the strongest across all conditions (ges ranging from 0.963 to 0.984), but the relative contribution of formulation varied considerably by bond type. Formulation effects were only modest for hydrogen bonds (ges = 0.367), larger for disulfide bonds (ges = 0.873), and largest for hydrophobic interactions (ges = 0.933), suggesting that compositional differences between hemp and oat proteins are most clearly expressed when hydrophobic interactions are disrupted. The disruption of hydrogen bonds and hydrophobic interactions showed the largest absolute increases in solubility, hinting at their importance in stabilizing protein structures in these systems. In contrast, disulfide bond disruption showed minimal increases, with solubility values remaining close to those observed under native conditions, suggesting a comparatively minor overall contribution of disulfide bonds to structural stabilization. Overall, while processing conditions were strongly associated with the solubility change across all single-bond conditions within this dataset, hydrophobic interactions appeared to be the bond type through which matrix composition exerted its strongest influence on protein structural behavior. However, it should be noted that the extraction approach used here reflects operational solubility rather than absolute structural quantification and therefore does not allow a strict ranking of bond importance.

A clear shift in the relative importance of treatment and formulation emerged as progressively more bond types were disrupted simultaneously. While single-bond conditions consistently showed stronger treatment-associated effects, dual-bond disruption reversed this pattern: formulation became the larger factor for hydrogen bonds × disulfide bonds (ges = 0.930 vs. 0.855) and, more markedly, for hydrogen bonds × hydrophobic interactions (ges = 0.961 vs. 0.645). The exception was disulfide bonds × hydrophobic interactions, where treatment retained a comparably large effect alongside formulation (ges = 0.944 vs. 0.959), suggesting this combination of interactions remains jointly governed by composition and processing rather than being formulation-dominant. Complete disruption of all interactions (solution 8) confirmed this trend most clearly, with formulation reaching its highest effect size across the entire dataset (ges = 0.975) and treatment dropping to its lowest (ges = 0.317), suggesting that when all bonds are broken, protein solubility is almost entirely associated with intrinsic differences between hemp and oat proteins.

Taken together, these results suggest that treatment, particularly through its effect on hydrogen bonds and hydrophobic interactions, may primarily affect protein conformation and aggregation in lightly to moderately disrupted conditions, whereas formulation may mainly affect the underlying bonding potential and structural resilience of the protein matrix, becoming the dominant factor once multiple interaction types are simultaneously removed. When considered alongside the amino acid and BA data, this suggests that protein structural breakdown during processing facilitates the release of free amino acids, but subsequent conversion into BA does not follow directly from substrate availability alone, pointing to the involvement of selective conversion processes whose mechanistic basis, whether enzymatic, microbial, or both, requires further characterization.

### 3.4. SDS-PAGE

**Figure 6:**
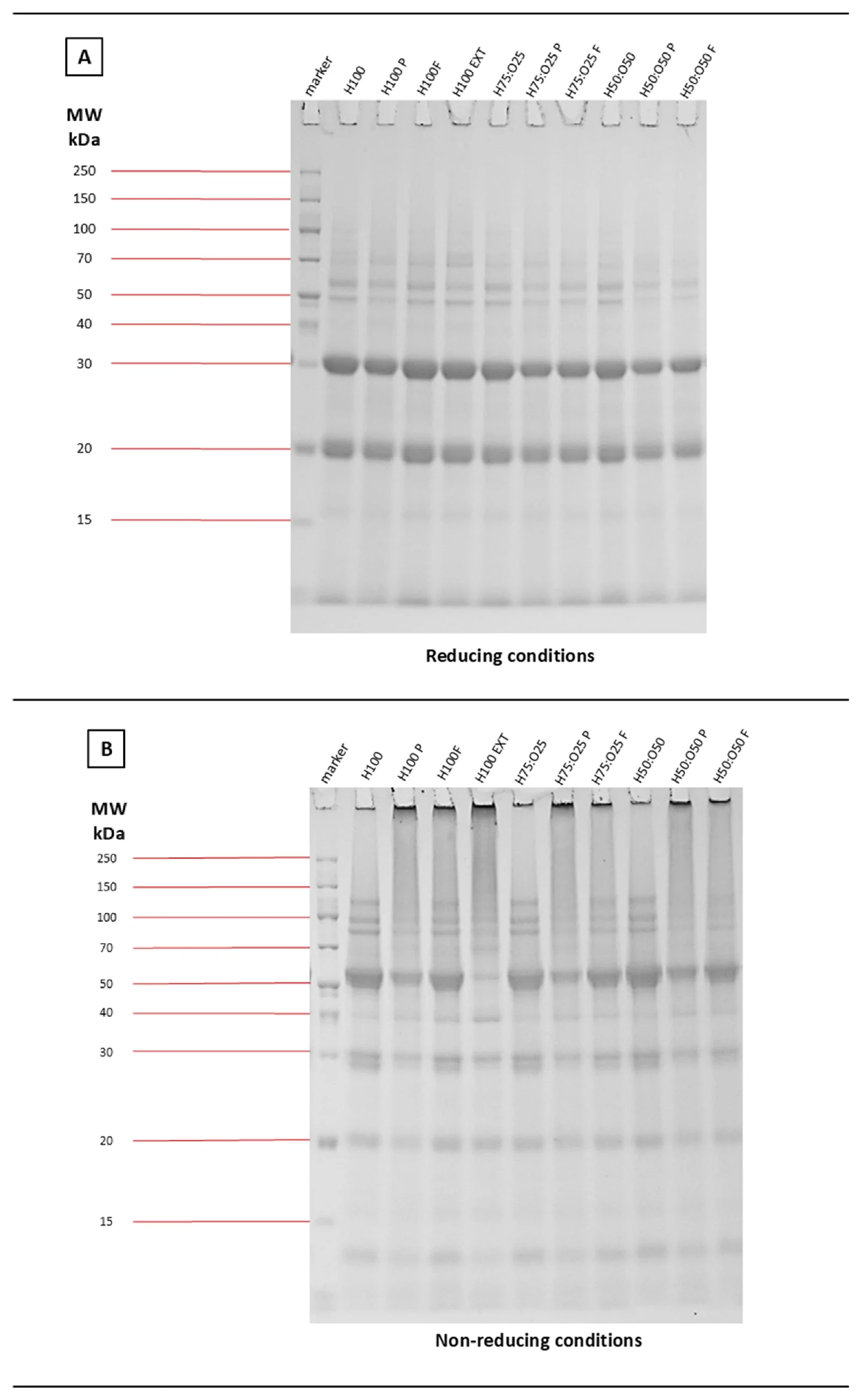
SDS-PAGE gel of combined hemp and oat press cake formulations (H100: hemp press cake, H75:O25: formulation 75% hemp press cake and 25% oat press cake, H50:O50: formulation 50% hemp press cake and 50% oat press cake, P: pasteurized, F: fermented, EXT: dry extruded) in reducing (**A**) and non-reducing conditions (**B**).

In reducing conditions (Figure 6A), the main bands were observed at around 20 and 30 kDa, with additional bands at approximately 50 kDa and slightly above 15 kDa. Wang et al. [53] reported major subunits of hemp protein isolates at approximately 48, 34, 20 and 18 kDa, with the 34 and 20 kDa fractions being the most abundant, which is consistent with the band pattern observed in the present study. Additional bands between 50–70 kDa and around 70 kDa were also detected, which may suggest incomplete reduction reaction, with some disulfide bonds remaining intact. Similarly, Docimo et al. [54], reported the bands at around 20 and 34 kDa corresponding to the β- and α-subunits of edestin.

In non-reducing conditions (Figure 6B), a dominant band was observed between 50 and 55 kDa, consistent with previous findings attributing this band (~52.5 kDa) to acid and basic subunits linked by disulfide bonds [53]. Additional bands at around at 30 kDa (double band) and 20 kDa were also visible, in agreement with earlier reports, although relative band intensities may differ due to methodological variations. Bands between 70 and 100 kDa and 100 and 150 kDa, as well as faint bands between 10 and 15 kDa, were also observed and may correspond to partial fragmentation or aggregation of edestin (≈310 kDa) induced by heat treatment. It should be noted that SDS-PAGE provides qualitative information on protein profiles, and therefore differences in band intensity should be interpreted with caution.

#### 3.4.1. Effects of Matrix Formulation

The band patterns of the H100, H75:O25, and H50:O50 samples subjected to the same treatment appeared largely similar. This suggests that the protein profile is mainly governed by the hemp fraction, which represents the dominant protein source in all formulations. Variations in band intensity across samples may reflect differences in total protein content rather than major changes in protein composition.

#### 3.4.2. Effects of Pasteurization, Fermentation and Extrusion

Under non-reducing conditions, changes in band patterns were observed after processing. Pasteurization was associated with a reduction in visible bands and an apparent accumulation of material at the top of the gel, suggesting the formation of insoluble, high-molecular-weight aggregates. This is consistent with previous reports showing heat-induced aggregation in hemp proteins [47].

These observations are in line with the formation of disulfide-linked aggregates through sulfhydryl–disulfide interchange reactions, as described by Tang et al. [55], and with the increased solubility observed under reducing conditions. The partial recovery observed after fermentation may be linked to structural rearrangements induced by pH changes and potentially by proteolytic activity, though the specific drivers were not characterized in this study.

It should also be noted that samples were dried after pasteurization and fermentation (oven drying at 60 °C for 24 h), which may have contributed to additional protein denaturation or aggregation and therefore influenced the observed band patterns.

The disappearance of high-molecular-weight bands (70–150 kDa) after pasteurization and their partial reappearance after fermentation may further reflect pH- and proteolysis-driven solubility changes. The pH decreased from approximately 6.5 in raw samples to around 5.2 after fermentation. Given that hemp globulins (mainly edestin) have isoelectric points between 4.5 and 5.9 [56,57,58], this shift towards more acidic conditions would generally be expected to decrease solubility. Proteolytic activity during fermentation may have contributed to partial resolubilization of the protein fraction, though this remains an inference consistent with the solubility trends observed in Section 3.3.9 rather than a directly confirmed mechanism.

### 3.5. Antinutritional Factor (Phytic Acid) Content

Within the given dataset, phytic acid content was strongly associated with both formulation and processing, with a pronounced interaction between the two factors (ges = 0.926). Overall, formulation showed a very large observed effect (ges = 0.994), as did treatment (ges = 0.815), suggesting that both composition and processing may influence phytic acid levels but not independently (Table A12).

**Figure 7:**
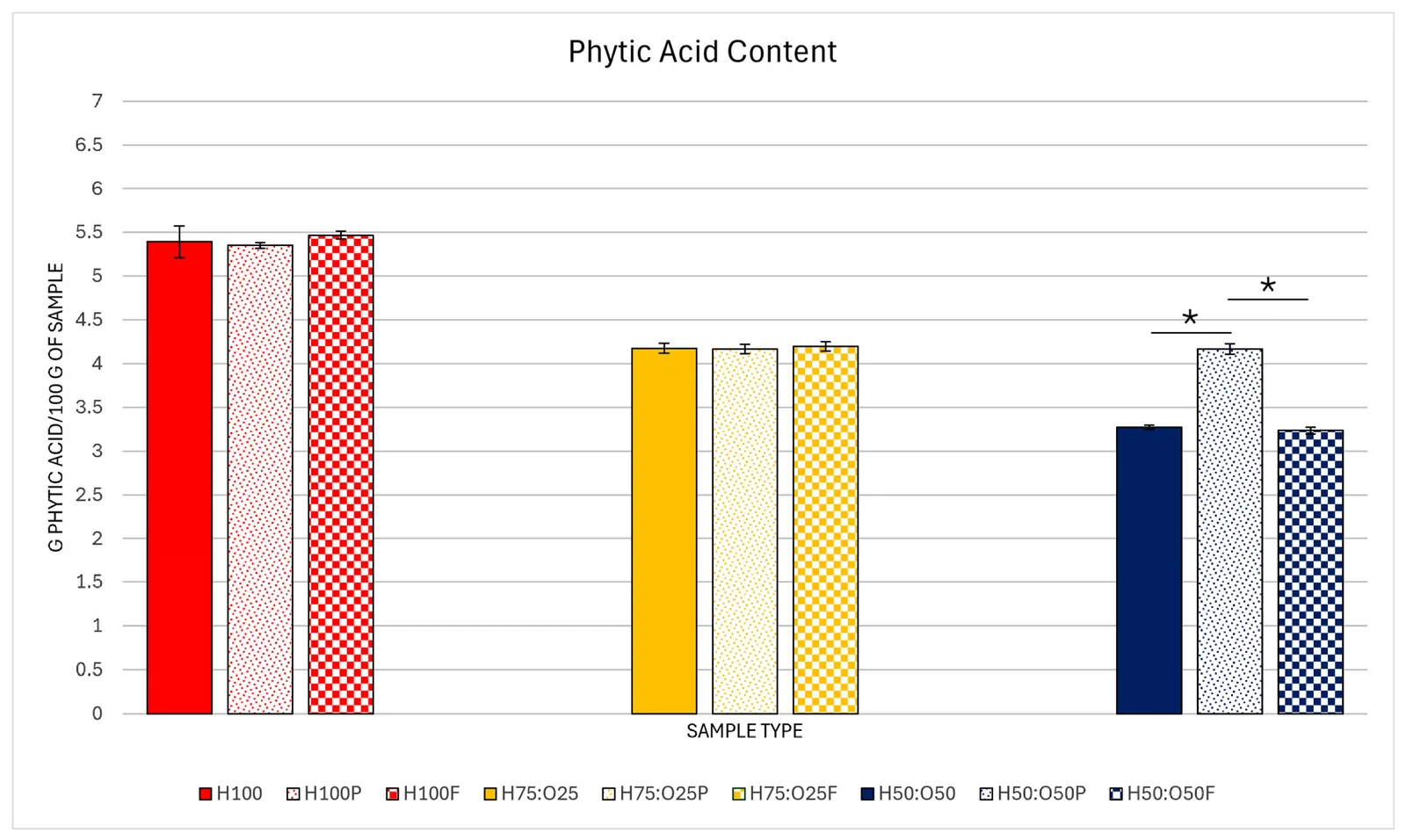
Phytic acid content of plant-based combined matrices calculated by phosphorus content. (H100: hemp press cake, H75:O25: formulation 75% hemp press cake and 25% oat press cake, H50:O50: formulation 50% hemp press cake and 50% oat press cake, P: pasteurized, F: fermented). The error bars represent the standard deviation (SD). The horizontal bars with a (*) symbol above indicate notable observed pairwise differences between samples of the same formulation.

As shown in Figure 7, across treatments, H100 showed stable phytic acid levels (approximately 5.35–5.47 g/100 g), with no notable differences between RAW, PAS and FER samples. Similarly, in H75:O25, no notable differences were observed between treatments, indicating the stability of phytic acid content across processing conditions in this formulation. In contrast, H50:O50 showed a treatment-associated pattern, where pasteurization was associated with notably higher phytic acid levels relative to RAW (from 3.27 to 4.17 g/100 g), while fermentation reduced them back to baseline values, with H50:O50F not showing notable differences from H50:O50 RAW.

From a treatment perspective, these effects were mainly associated exclusively with the H50:O50 mixed formulation. Treatment showed a large effect size in this formulation (ges = 0.943), whereas no notable changes were observed in H100 or H75:O25. This suggests that the overall interaction arises from a formulation-specific response rather than a uniform treatment effect across samples.

The increase in phytic acid after pasteurization in H50:O50 may be explained by thermal inactivation of endogenous phytases, which are known to be present in both oat and hemp matrices due to their ability to reduce phytic acid during germination and processing [59,60]. Since phytases are heat-sensitive, thermal treatment likely reduces their activity, limiting endogenous phytic acid degradation [61]. In contrast, fermentation was associated with reduced phytic acid levels, which may reflect the restoration of phytase activity through fermentation-associated processes, potentially including microbial phytase contribution, though this mechanism was not directly characterized in the present study and requires further investigation.

Overall, these results suggest that, within this dataset, phytic acid behavior is highly formulation-dependent, with treatment effects confined to the most oat-rich formulation. The absence of treatment effects in H100 and H75:O25 suggests that a sufficient proportion of oat press cake is required to observe processing-responsive phytate dynamics, possibly related to differences in endogenous phytase content or activity between hemp and oat matrices.

Although some effects of formulation and processing were observed for phytic acid, the biological relevance of these differences should be interpreted with caution. Phytic acid is not considered harmful at dietary levels typically encountered in plant-based foods, and no established intake threshold exists for toxicity. In typical diets, phytate intake can range from a few hundred milligrams per day in mixed diets to over 1 g/day in plant-rich diets without adverse effects being directly attributed to its presence alone [62]. Its main nutritional relevance lies in its ability to reduce mineral bioavailability, particularly iron and zinc, rather than in absolute concentration per se [63]. In the present study, phytic acid levels remained relatively high across all samples, suggesting that although fermentation and blending may be able to modulate its content, the overall reduction may be insufficient to substantially alter mineral bioavailability without additional processing strategies.

### 3.6. Techno-Functional Parameters (WHC and OHC)

Water-holding capacity (WHC) and oil-holding capacity (OHC) (Table 5) are key functional attributes that influence the texture, juiciness, and processability of plant-based meat analogues. In this study, both properties were affected by formulation and processing, although the strength and nature of the effects differed between parameters.

WHC showed notable variations across samples (Welch ANOVA, see Table A13), suggesting formulation-associated differences (Appendix B, Table A13). Values ranged from 0.94 to 2.28 g water/g sample, with the highest WHC observed in H100F (2.28 ± 0.11 g/g) and H100 (1.95 ± 0.07 g/g). The addition of oat press cake generally showed a downward pattern of WHC within RAW and FER treatments. This may be related to differences in dietary fiber composition and structure. Hemp press cakes contain substantial fiber fractions capable of water binding and swelling [64], whereas oat β-glucans, although highly hydrophilic, are mainly concentrated in the bran fraction and are less represented in press cake material [65]. As a result, blending seems to be associated with a reduction in the overall water-binding capacity of the system. PAS samples showed the lowest WHC values overall, suggesting strong effects of processing, although the exact mechanisms were not characterized.

OHC showed notable effects of formulation (ges = 0.822), treatment (ges = 0.698), and their interaction (ges = 0.494), suggesting that both formulation and processing may influence lipid retention but not independently (Appendix B, Table A14). Pure hemp samples exhibited the highest OHC values compared to combined formulations, hinting at a strong contribution of hemp-derived structural components to lipid binding. Fermentation seemed to further modify OHC within this dataset, but its effect depended on formulation, as reflected by the notable interaction effect size. In general, fermentation appeared to improve OHC in pure hemp, while mixed formulations showed more limited or variable responses.

Principal effects analysis (Table A14) provided further insights into the nature of the factor interaction for OHC. Observed treatment effects were strongest in pure hemp (H100; ges = 0.693) and progressively decreased in mixed formulations, suggesting that oat incorporation reduces the responsiveness of oil-holding capacity to processing. Conversely, differences between formulations were most pronounced after fermentation (ges = 0.817), suggesting that fermentation amplifies matrix-dependent functional properties within this dataset. In contrast, pasteurization showed weaker sample differentiation across formulations. These results suggest that OHC is strongly associated with composition-dependent response to processing rather than by either factor alone.

Overall, these results suggest that WHC is affected by formulation and processing, although the Welch ANOVA test did not allow estimation of their separate effects. On the other hand, OHC is jointly modified by formulation and processing. The interaction observed for OHC points at functional properties of lipid retention being matrix-dependent and sensitive to fermentation in a composition-specific manner.

### 3.7. Particle Size Distribution

Particle size distribution is a useful parameter to understand the properties of the raw materials and their suitability for different applications. In the case of press cakes, further processing, such as extrusion cooking, can closely depend on the characteristics and behavior of the particles that make them up. Three particle size parameters were collected in this study, the fine particle threshold (Dv10), the median particle size (Dv50) and the coarse particle threshold (Dv90) (Table 6).

The median particle size, which represents the average size of particles in the sample, was on average similar for the untreated formulations. On the other hand, pasteurization and fermentation steps were associated with increases in the median particle size in all cases, which suggests particle aggregation. The fermentation of plant materials has previously been shown to degrade certain compounds, such as wall polysaccharides [66], which could suggest smaller particle sizes. However, within this dataset, the contrary is observed, as pasteurized and fermented samples consistently showed higher median particle sizes than their untreated counterparts. While the current knowledge on the relationship between fermentation and particle size is limited, previous research has shown that moisture levels can induce higher particle and fiber sizes in plant materials [67]. We hypothesize that the higher humidity conditions of the pasteurization and fermentation processes influenced aggregation, which resulted in larger median particle size. It should also be noted that all processed samples were oven-dried at 60 °C for 24 h prior to analysis. This drying step may have contributed independently to particle aggregation and compaction, and its effect cannot be fully decoupled from those of pasteurization and fermentation in the current experimental design. Future studies should consider analyzing particle size before and after drying to isolate the contribution of this step.

The fine particle threshold decreased along with the increase in OPC in the compositions for non-fermented samples; however, this was not the case for the pasteurized and fermented samples. The OPC contains finer particles (Dv10 = 7.97 µm), which may have influenced the fine particle threshold of the untreated samples. However, its influence was possibly minimized with the change in conditions induced by the pasteurization and fermentation processes.

In the case of the coarse particle threshold, both the pure oat and the pure hemp showed similar values, which may also explain the absence of variation for the unfermented combinations. However, pasteurization and fermentation seemed to have a bigger aggregation effect on the combined matrices than on the pure hemp sample.

Overall, within this dataset, the combined formulation composition was associated with observable differences in the fine particle threshold, whereas the coarse particle threshold is mainly affected by the processing method. The median particle size is in turn affected by both parameters, highlighting the need to take them both into consideration in order to obtain the desired characteristics for further applications of the raw materials.

## 4. Conclusions

This study investigated whether lactic acid fermentation would modulate the structural, nutritional, and techno-functional properties of combined hemp–oat press cake matrices in a formulation-dependent manner. The observed patterns were consistent with this hypothesis for several parameters, though the single-batch design limits the generalizability of these findings, and confirmation through independently replicated experiments is required. The relative importance of formulation and processing appeared to be parameter-dependent: formulation was associated with the largest observed differences in total amino acid content, phytic acid levels, biogenic amine accumulation, and protein solubility under multi-bond disruption conditions, while treatment was associated with greater variation in free amino acid content, protein solubility under phosphate buffer and single-reagent extraction conditions. Protein carbonyl content was associated with both factors, with the formulation effect exceeding the treatment effect and treatment-associated differences detected only in the mixed formulations. Particle-size measurements showed clear descriptive differences between the RAW and processed samples, but formulation and treatment effects were not statistically decomposed. Biogenic amine profiles showed large observed formulation, treatment, and interaction effects relative to the analytical variability within the batch examined.

Protein interaction analysis suggested that treatment was associated with the largest observed differences in solubility across single-bond disrupting conditions, with hydrogen bonds and hydrophobic interactions appearing as the main structural stabilizing forces, while formulation-associated differences became more pronounced only upon complete disruption of all interactions. Disulfide bonds showed a comparatively limited structural contribution. SDS-PAGE patterns were consistent with hemp protein dominance across all formulations, with pasteurization associated with high-molecular-weight aggregate formation and fermentation associated with partial solubility restoration, possibly through pH-mediated changes, though the mechanistic basis warrants further investigation. Particle size increased after both processing steps, consistent with moisture- and heat-driven aggregation, though the contribution of the post-processing drying step cannot be fully excluded.

From a nutritional perspective, proteolysis and biogenic amine formation appeared shaped by both factors, though the specific enzymatic and microbial mechanisms were not directly characterized. Protein oxidation remained relatively high across all conditions, with reductions observed only in combined formulations after fermentation; a similar pattern was observed for phytic acid content, with treatment-associated differences confined to H50:O50. Additional or more targeted strategies may be required to substantially improve these parameters. Techno-functional properties appeared also dependent on both factors, with WHC showing a consistent descriptive decrease with increasing oat proportion within each treatment, possibly related to fiber content, and a consistent decrease associated with pasteurization, although the Welch ANOVA did not allow separate formulation and treatment effects to be estimated. OHC showed influence of both factors and their interaction, with H100 exhibiting the highest values across treatments.

Across the measured parameters, no single formulation was superior in all respects within this dataset. H100 showed the highest protein content, WHC, and OHC but also generally higher phytic acid and total biogenic amine contents, while H50:O50 showed the lowest phytic acid values in RAW and FER samples and a pronounced fermentation-associated increase in free amino acids but had lower protein content, WHC, and OHC. H75:O25 maintained a higher protein content than H50:O50 and showed several potentially favorable within-batch patterns, including a low protein carbonyl content after fermentation and free amino acid release comparable to H50:O50, which may therefore represent a promising compromise among the formulations examined. Future work should include a pure OPC reference formulation to fully elucidate individual matrix contributions, a systematic gradient design and independently prepared batches per condition to allow robust statistical inference and identify optimal blending proportions for specific functional or nutritional targets.

From an application perspective, these findings suggest that lactic acid fermentation of combined press cake matrices may provide a means of modifying protein extractability, particle-size characteristics, amino acid profiles, and techno-functional properties, although trade-offs between protein oxidation, phytic acid content, protein content, and functional performance must be considered. These application-oriented interpretations should remain provisional until the observed patterns are reproduced in independently prepared batches.

Future work should focus on optimizing fermentation conditions, linking the observed changes in protein extractability and composition to protein digestibility and mineral bioavailability in physiological models, and characterizing the associated microbial, enzymatic, and metabolite profiles. Only one independently prepared processing batch per formulation × treatment combination was analyzed, while the duplicate or triplicate measurements represented analytical replicates. Consequently, the statistical results describe within-batch patterns and analytical variability but do not estimate between-batch variation. Future studies should analyze multiple independently prepared batches per condition to support statistical inference. Particle size should also be evaluated before and after drying to distinguish the contribution of this step from those of pasteurization and fermentation. Moreover, the utilization of fermented press cake matrices as raw materials for high-moisture extrusion represents a promising application for further investigation.

## Figures and Tables

**Table 1:** Nutritional composition of press cake formulations. (H100: hemp press cake, H75:O25: formulation 75% hemp press cake and 25% oat press cake, H50:O50: formulation 50% hemp press cake and 50% oat press cake, P: pasteurized, F: fermented).

Component (Dry Matter %)	H100	H100P	H100F	H75:O25	H75:O25P	H75:O25F	H50:O50	H50:O50P	H50:O50F
Protein (%)	45.7	47.3	47.4	38.4	39.7	39.5	30.6	30.7	31.2
Carbohydrates excluding fibers (%)	3.61	0.40	1.09	17.3	15.2	19.6	35.0	33.1	35.5
Fibers (%)	32.0	31.6	31.2	27.3	27.3	22.6	19.2	21.1	17.0
Fat (%)	10.7	12.2	11.4	10.4	10.9	11.2	9.88	10.0	10.7
Ash	8.00	8.45	8.95	6.73	6.89	7.17	5.32	5.15	5.62

Measurements performed in duplicate by an accredited laboratory. Only the mean value is presented.

**Table 2:** Two-factor ANOVA and Scheirer–Ray–Hare test results for total amino acid content across hemp–oat press cake formulations subjected to three processing treatments (RAW, PAS, FER). Effects of formulation, treatment, and their interaction are reported for each amino acid and for the sum of total amino acids.

Amino Acid	Formulation			Treatment			Interaction		
	F/H	*p*-Value	Ges	F/H	*p*-Value	Ges	F/H	*p*-Value	Ges
asx	120.091	<0.001 *	0.964	65.476	<0.001 *	0.936	3.615	0.051	0.616
glx	110.005	<0.001 *	0.961	109.838	<0.001 *	0.961	8.518	0.004 *	0.791
ser+	9.871	0.007 *	-	2.895	0.142	-	1.778	0.777	-
gly	36.731	<0.001 *	0.891	11.909	0.003 *	0.726	8.802	0.004 *	0.796
his+	1.368	0.504	-	14.363	<0.001 *	-	0.585	0.965	-
thr+	6.327	0.042 *	-	2.211	0.331	-	7.216	0.125	-
ala	199.067	<0.001 *	0.978	222.405	<0.001 *	0.980	13.946	<0.001 *	0.861
arg	105.281	<0.001 *	0.959	19.177	<0.001 *	0.810	5.603	0.015 *	0.713
pro	3.301	0.084	0.423	19.63	<0.001 *	0.814	16.143	<0.001 *	0.878
cys+	0.082	0.960	-	11.474	0.003 *	-	4.690	0.321	-
tyr	24.531	<0.001 *	0.845	7.000	0.015 *	0.609	3.642	0.05 *	0.618
val	44.839	<0.001 *	0.909	0.537	0.602	0.107	12.092	0.001 *	0.843
met	0.283	0.760	0.059	23.063	<0.001 *	0.837	1.69	0.236	0.429
lys	199.860	<0.001 *	0.978	50.677	<0.001 *	0.918	3.635	0.05 *	0.618
ile	151.836	<0.001 *	0.971	18.918	<0.001 *	0.808	17.693	0.001 *	0.887
leu	146.030	<0.001 *	0.970	12.960	0.002 *	0.742	6.469	0.01 *	0.742
phe	92.692	<0.001 *	0.954	0.044	0.957	0.010	26.952	<0.001 *	0.923
SUM	121.444	<0.001 *	0.979	21.961	<0.001 *	0.830	20.347	<0.001 *	0.900

asx = aspartic acid + asparagine; glx = glutamic acid + glutamine. F: test statistic for two-factor ANOVA; H: test statistic for Scheirer–Ray–Hare non-parametric test, applied where normality assumptions were not met (indicated by +). ges: generalized eta-squared, reported as effect size for ANOVA only; not applicable for Scheirer–Ray–Hare results (-). Post hoc pairwise comparisons were performed using Tukey’s HSD for ANOVA and Dunn’s test with Bonferroni correction for Scheirer–Ray–Hare. Asterisks (*) indicate *p* < 0.05, reported for descriptive purposes only. Values for cys and met may be partially underestimated due to standard acid hydrolysis degradation.

**Table 3:** Two-factor ANOVA and Scheirer–Ray–Hare test results for free amino acid content across hemp–oat press cake formulations subjected to three processing treatments (RAW, PAS, FER). Effects of formulation, treatment, and their interaction are reported for each amino acid and for the sum of free amino acids.

Amino Acid	Formulation			Treatment			Interaction		
	F/H	*p*-Value	Ges	F/H	*p*-Value	Ges	F/H	*p*-Value	Ges
asp+	3.135	0.209	-	11.661	0.003 *	-	1.485	0.829	-
asn	8.006	0.01 *	0.64	64.57	<0.001 *	0.935	27.357	<0.001 *	0.924
glu+	0.222	0.895	-	14.749	<0.001 *	-	1.520	0.823	-
ser+	0.105	0.949	-	10.889	0.004 *	-	3.497	0.478	-
gly	47.379	<0.001 *	0.913	90.356	<0.001 *	0.953	44.178	<0.001 *	0.952
his	4.781	0.038 *	0.515	29.837	<0.001 *	0.869	15.409	<0.001 *	0.873
thr	105.648	<0.001 *	0.959	179.205	<0.001 *	0.976	47.93	<0.001 *	0.955
ala	55.135	<0.001 *	0.925	244.339	<0.001 *	0.982	81.685	<0.001 *	0.973
arg	203.378	<0.001 *	0.978	231.62	<0.001 *	0.981	272.682	<0.001 *	0.992
pro	31.768	<0.001 *	0.876	136.117	<0.001 *	0.968	3.945	0.041 *	0.637
cys	22.175	<0.001 *	0.831	79.927	<0.001 *	0.947	17.421	<0.001 *	0.886
tyr	0.032	0.969	0.007	14.587	0.002 *	0.764	0.939	0.484	0.294
val	82.269	<0.001 *	0.948	125.033	<0.001 *	0.965	52.93	<0.001 *	0.959
met	136.413	<0.001 *	0.968	288.092	<0.001 *	0.985	114.276	<0.001 *	0.981
lys	74.874	<0.001 *	0.943	131.997	<0.001 *	0.967	42.717	<0.001 *	0.950
ile	27.735	<0.001 *	0.860	57.623	<0.001 *	0.928	23.955	<0.001 *	0.914
leu	321.46	<0.001 *	0.986	539.01	<0.001 *	0.992	190.106	<0.001 *	0.988
phe	64.57	<0.001 *	0.935	149.445	<0.001 *	0.971	53.189	<0.001 *	0.959
trp	0.667	0.537	0.129	1.38	0.3	0.235	0.433	0.782	0.161
SUM	6.421	0.019 *	0.588	119.849	<0.001 *	0.964	23.794	<0.001 *	0.914

F: test statistic for two-factor ANOVA; H: test statistic for Scheirer–Ray–Hare non-parametric test, applied where normality assumptions were not met (indicated by +). ges: generalized eta-squared, reported as effect size for ANOVA only; not applicable for Scheirer–Ray–Hare results (-). Post hoc pairwise comparisons were performed using Tukey’s HSD for ANOVA and Dunn’s test with Bonferroni correction for Scheirer–Ray–Hare. Asterisks (*) indicate *p* < 0.05, reported for descriptive purposes only.

**Table 4:** Two-factor ANOVA results for biogenic amine content across hemp–oat press cake formulations subjected to three processing treatments (RAW, PAS, FER). Effects of formulation, treatment, and their interaction are reported for each biogenic amine and for total biogenic amine content.

Biogenic Amine	Formulation			Treatment			Interaction		
	F	*p*-Value	Ges	F	*p*-Value	Ges	F	*p*-Value	Ges
Tyramine	124.294	<0.001 *	0.965	807.105	<0.001 *	0.994	119.569	<0.001 *	0.982
Putrescine	1183.523	<0.001 *	0.996	164.271	<0.001 *	0.973	109.691	<0.001 *	0.980
Spermidine	34,653.52	<0.001 *	1.000	32,969.49	<0.001 *	1.000	6784.46	<0.001 *	1.000
Spermine	2481.873	<0.001 *	0.980	4415.999	<0.001 *	0.999	237.380	<0.001 *	0.991
Total	10,806.270	<0.001 *	1.000	259.724	<0.001 *	0.983	507.349	<0.001 *	0.996

F: test statistic for two-factor ANOVA. ges: generalized eta-squared, reported as the primary descriptor of observed effect magnitude. F-statistics and *p*-values are reported for descriptive purposes only and should not be interpreted as inferential hypothesis tests given the single-batch design. Post hoc pairwise comparisons were performed using Tukey’s HSD. Asterisks (*) indicate *p* < 0.05. Histamine and cadaverine were excluded from the statistical analysis due to near-zero concentrations across all samples.

**Table 5:** WHC and OHC of press cake formulations (H100: hemp press cake, H75:O25: formulation 75% hemp press cake and 25% oat press cake, H50:O50: formulation 50% hemp press cake and 50% oat press cake, P: pasteurized, F: fermented).

Sample	Blend Ratios		Treatment	WHC	OHC
	HPC	OPC			
H100	100	0	RAW	1.95 ± 0.07 ^a^	1.21 ± 0.09 ^a^
H100P	100	0	PAS	0.95 ± 0.01 ^b^	1.54 ± 0.04 ^b^
H100F	100	0	FER	2.28 ± 0.11 ^c^	1.66 ± 0.17 ^b^
H75:O25	75	25	RAW	1.68 ± 0.07 ^a^	0.84 ± 0.11 ^a^
H75:O25P	75	25	PAS	0.94 ± 0.03 ^b^	1.27 ± 0.08 ^b^
H75:O25F	75	25	FER	1.71 ± 0.07 ^a^	0.87 ± 0.14 ^a^
H50:O50	50	50	RAW	1.41 ± 0.04 ^a^	0.90 ± 0.17 ^a^
H50:O50P	50	50	PAS	0.99 ± 0.02 ^b^	1.22 ± 0.05 ^b^
H50:O50F	50	50	FER	1.47 ± 0.07 ^a^	0.86 ± 0.13 ^a^

Values with different letter exponents in the same triplet indicate notably different observed values based on post hoc pairwise comparisons (Games–Howell and Tukey tests for WHC and OHC, respectively).

**Table 6:** Particle size analysis of plant-based combined matrices (H100: hemp press cake, H75:O25: formulation 75% hemp press cake and 25% oat press cake, H50:O50: formulation 50% hemp press cake and 50% oat press cake, P: pasteurized, F: fermented, OPC: oat press cake).

Sample	Blend Ratios		Treatment	Dv10 (μm)	Dv50 (μm)	Dv90 (μm)
	HPC	OPC				
H100	100	0	RAW	33.4 ± 0.331 ^a^	202 ± 1.94 ^a^	448 ± 6.02 ^a^
H100P	100	0	PAS	92.6 ± 1.23 ^b^	338 ± 3.25 ^b^	655 ± 3.20 ^b^
H100F	100	0	FER	114 ± 3.80 ^c^	439 ± 6.69 ^c^	850 ± 11.7 ^c^
H75:O25	75	25	RAW	19.7 ± 0.364 ^a^	178 ± 1.77 ^a^	435 ± 11.1 ^a^
H75:O25P	75	25	PAS	87.9 ± 8.57 ^b^	428 ± 25.1 ^b^	901 ± 52.4 ^b^
H75:O25F	75	25	FER	118 ± 1.38 ^c^	461 ± 3.28 ^b^	857 ± 6.37 ^b^
H50:O50	50	50	RAW	15.9 ± 0.113 ^a^	174 ± 4.51 ^a^	452 ± 11.8 ^a^
H50:O50P	50	50	PAS	131 ± 16.4 ^b^	518 ± 36.6 ^b^	1050 ± 34.5 ^b^
H50:O50F	50	50	FER	81.8 ± 2.48 ^c^	531 ± 12.9 ^b^	1090 ± 24.5 ^b^
OPC	0	100	RAW	7.97 ± 0.02	97 ± 7.6	410 ± 7.65

Values with different letter exponents in the same triplet indicate notably different observed values based on post hoc pairwise comparisons (Tukey test). Oat press cake (OPC) particle size is given as a reference.

## Data Availability

The original data presented in the study are openly available in Zenodo at 10.5281/zenodo.20049050.

## Supplementary Materials

The following supporting information can be downloaded at: https://www.mdpi.com/article/10.3390/foods15183357/s1, Table S1: LOQ and LOD calculator.

## Abbreviations

The following abbreviations are used in this manuscript:

HPC	Hemp press cake
OPC	Oat press cake
SDS-PAGE	Sodium dodecyl sulfate–polyacrylamide gel electrophoresis
RAW	Untreated samples
PAS	Pasteurized samples
FER	Fermented samples
HPLC	High-performance liquid chromatography
UHPLC	Ultra-high-performance liquid chromatography
UFLC	Ultra-fast liquid chromatography
OPA	O-phthalaldehyde
FMOC	9-fluorenylmethyl chloroformate
MERC	Mercatopropionic acid
TCA	Trichloroacetic acid
SDS	Sodium dodecyl sulfate
DNPH	2,4-dinitrophenylhydrazine
NSP	Native state protein
HB	Hydrogen bonds
DISB	Disulfide bonds
HYDX	Hydrophobic interactions
DTT	Dithiothreitol
BSA	Bovine serum albumin
MOPS	3-(N-morpholino)propanesulfonic acid
WHC	Water-holding capacity
OHC	Oil-holding capacity
ANOVA	Analysis of variance
PCA	Principal component analysis
PC	Principal component
BA	Biogenic amines

## Appendix A. Result Tables—Means, Standard Deviations and Post Hoc Comparisons

**Table A1:** Total amino acid content (mg/100 g sample) of hemp–oat press cake formulations across three processing treatments (RAW, PAS, FER). Values are expressed as mean ± standard deviation.

Total Amino Acids (mg/100 g Sample)	Samples								
	H100	H100P	H100F	H75:O25	H75:O25P	H75:O25F	H50:O50	H50:O50P	H50:O50F
asx	5864 ± 144 ^a^	4767 ± 214 ^b^	5315 ± 431 ^ab^	4804 ± 293 ^a^	2927 ± 174 ^b^	4590 ± 234 ^a^	3845 ± 13 ^a^	2630 ± 118 ^b^	3603 ± 39 ^a^
glx	9207 ± 428 ^a^*	7227 ± 62 ^b^*	8270 ± 588 ^c^*	7968 ± 261 ^a^	4677 ± 130 ^b^	7446 ± 201 ^a^	6288 ± 118 ^a^	5033 ± 16 ^b^	6496 ± 2 ^a^
ser^+^	2410 ± 145 ^a^	2110 ± 314 ^a^	2135 ± 110 ^a^	2029 ± 29 ^a^	1316 ± 4 ^a^	2133 ± 11 ^a^	1665 ± 30 ^a^	1195 ± 39 ^a^	1578 ± 12 ^a^
gly	1642 ± 75 ^a^	1686 ± 204 ^a^	1525 ± 121 ^a^	1487 ± 4 ^a^	1129 ± 7 ^b^	1689 ± 52 ^a^	1153 ± 32 ^ab^	1009 ± 17 ^a^*	1364 ± 83 ^b^*
his^+^	221 ± 11 ^a^	1408 ± 875 ^a^	359 ± 8 ^a^	261 ± 16 ^a^	990 ± 81 ^a^	776 ± 3 ^a^	613 ± 23 ^a^	730 ± 105 ^a^	174 ± 20 ^a^
thr^+^	1363 ± 113 ^a^	2355 ± 672 ^a^	1468 ± 192 ^a^	1398 ± 10 ^a^	1002 ± 33 ^a^	1660 ± 22 ^a^	1065 ± 18 ^a^	889 ± 3 ^a^	1332 ± 46 ^a^
ala	1954 ± 0 ^a^	1594 ± 20 ^b^	1861 ± 104 ^a^	1731 ± 35 ^a^	1109 ± 10 ^b^	1790 ± 20 ^a^	1362 ± 52 ^a^*	1009 ± 36 ^b^	1512 ± 25 ^c^*
arg	5049 ± 393 ^a^	4696 ± 577 ^a^	4606 ± 17 ^a^	4112 ± 6 ^a^	2833 ± 28 ^b^	4390 ± 154 ^a^	2902 ± 96 ^a^*	2195 ± 146 ^b^*	3006 ± 118 ^ab^
pro	777 ± 245 ^a^	821 ± 76 ^a^	459 ± 24 ^a^	468 ± 156 ^a^	532 ± 55 ^a^	1472 ± 60 ^b^	351 ± 118 ^a^	446 ± 49 ^a^	1068 ± 249 ^b^
cys^+^	225 ± 16 ^a^	760 ± 257 ^a^	371 ± 11 ^a^	302 ± 58 ^a^	371 ± 36 ^a^	464 ± 6 ^a^	287 ± 26 ^a^	438 ± 42 ^a^	425 ± 12 ^a^
tyr	1595 ± 98 ^a^*	1237 ± 133 ^b^	1345 ± 138 ^b^*	1350 ± 8 ^a^	1204 ± 32 ^a^	1277 ± 57 ^a^	1094 ± 28 ^a^	1136 ± 42 ^a^	1024 ± 17 ^a^
val	2447 ± 465 ^a^*	3151 ± 55 ^b^*	2137 ± 43 ^a^	2227 ± 78 ^a^	1386 ± 5 ^b^*	1999 ± 35 ^a^*	1487 ± 248 ^a^	1404 ± 239 ^a^	1676 ± 42 ^a^
met	677 ± 261 ^a^	988 ± 333 ^a^	690 ± 211 ^a^	369 ± 15 ^a^	1227 ± 172 ^b^*	535 ± 8 ^a^*	536 ± 100 ^a^*	1216 ± 124 ^b^*	441 ± 72 ^a^
lys	2428 ± 104 ^a^*	2328 ± 45 ^ab^	2138 ± 112 ^b^*	1987 ± 56 ^a^	1496 ± 21 ^b^	1338 ± 11 ^b^	1625 ± 145 ^a^	1385 ± 102 ^a^*	1121 ± 40 ^b^*
ile	1731 ± 14 ^a^	2291 ± 105 ^b^	1630 ± 163 ^a^	1480 ± 35 ^a^	1302 ± 49 ^a^	1328 ± 14 ^a^	1193 ± 24 ^a^	1248 ± 60 ^a^	1121 ± 24 ^a^
leu	3568 ± 145 ^a^*	3650 ± 48 ^a^	3124 ± 306 ^b^*	3004 ± 53 ^a^*	2395 ± 39 ^b^	2647 ± 31 ^b^*	2404 ± 20 ^a^	2259 ± 96 ^a^	2151 ± 13 ^a^
phe	2031 ± 60^a^	2231 ± 54 ^a^	1644 ± 155 ^b^	1614 ± 90 ^a^*	1435 ± 31 ^a^	1826 ± 23 ^b^*	1353 ± 61 ^a^	1363 ± 40 ^a^	1526 ± 12 ^a^
SUM	43,265 ± 2054 ^a^*	43,300 ± 97 ^a^*	39,079 ± 2300 ^b^*	36,592 ± 1023 ^a^	27,331 ± 831 ^b^	37,362 ± 142 ^a^	28,905 ± 52 ^ab^	25,583 ± 519 ^b^*	29,935 ± 762 ^a^*

asx = aspartic acid + asparagine; glx = glutamic acid + glutamine. Different lowercase letters within the same sample row indicate notable observed differences between treatments as determined by Tukey’s HSD post hoc test; these comparisons are reported for descriptive purposes only given the single-batch design. Asterisks (*) denote comparisons where 0.01 < *p* < 0.05; unmarked letter differences indicate *p* ≤ 0.01; (+) indicates amino acids for which the Scheirer–Ray–Hare non-parametric test was applied due to violation of normality assumptions, with post hoc comparisons performed using Dunn’s test with Bonferroni correction; all other amino acids were analyzed by two-factor ANOVA. HPC: hemp press cake; OPC: oat press cake; RAW: untreated (H100, H75:O25, H50:O50); PAS: pasteurized (H100P, H75:O25P, H50:O50P); FER: fermented (H100F, H75:O25F, H50:O50F). Values for cys and met may be partially underestimated due to standard acid hydrolysis degradation.

**Table A2:** Free amino acid content (mg/100 g sample) of hemp–oat press cake formulations across three processing treatments (RAW, PAS, FER). Values are expressed as mean ± standard deviation.

Free Amino Acids (mg/100 g Sample)	Samples								
	H100	H100P	H100F	H75:O25	H75:O25P	H75:O25F	H50:O50	H50:O50P	H50:O50F
asp^+^	96.3 ± 0.4 ^a^	44.9 ± 0.1 ^a^	93.3 ± 0.8 ^a^	74.6 ± 17.7 ^a^	8.9 ± 1.1 ^a^	92.4 ± 2.1 ^a^	80.4 ± 4.2 ^a^	8.1 ± 0.4 ^a^	85.9 ± 1.8 ^a^
asn	56.5 ± 8.8 ^a^	38.6 ± 0.3 ^ab^	18.2 ± 0.3 ^b^	52.9 ± 15.9 ^a^	2.8 ± 0.3 ^b^	52.3 ± 1.2 ^a^	64.4 ± 10.3 ^a^*	3.4 ± 0.2 ^b^	85.2 ± 3.9 ^c^*
glu^+^	213.7 ± 8.7 ^a^	125.3 ± 1.9 ^a^	19.6 ± 2.6 ^a^	149.0 ± 27.1 ^a^	46.9 ± 0.2 ^a^	22.0 ± 1.7 ^a^	142.0 ± 1.4 ^a^	40.0 ± 0.7 ^a^	38.9 ± 1.6 ^a^
ser^+^	11.8 ± 10.4 ^a^	11.4 ± 0.8 ^a^	13.2 ± 0.4 ^a^	14.7 ± 7.7 ^a^	1.63 ± 0.02 ^a^	36.8 ± 0.4 ^a^	17.1 ± 8.5 ^a^	1.87 ± 0.04 ^a^	33.4 ± 1.7 ^a^
gly	3.3 ± 1.7 ^a^	5.27 ± 0.02 ^a^	2.6 ± 1.0 ^a^	3.5 ± 2.8 ^a^	1.8 ± 0.2 ^a^	13.4 ± 0.3 ^b^	6.9 ± 3.8 ^a^	2.1 ±0.1 ^a^	31.3 ± 1.8 ^b^
his	5.3 ± 5.6 ^a^	17.5 ± 0.1 ^b^	4.8 ± 4.9 ^a^	4.2 ± 4.5 ^a^	10.6 ± 1.1 ^a^*	22.4 ± 1.1 ^b^*	4.5 ± 4.3 ^a^	9.97 ± 0.02 ^a^	31.0 ± 1.6 ^b^
thr	9.7 ± 3.5 ^a^	8.8 ± 2.5 ^a^	12.0 ± 5.3 ^a^	15.1 ± 2.7 ^a^	9.7 ± 3.5 ^a^	39.3 ± 1.6 ^b^	26.2 ± 2.2 ^a^	9.4 ± 0.1 ^b^	66.96 ± 0.04 ^c^
ala	52.5 ± 6.9 ^a^	32.6 ± 5.8 ^b^	9.0 ± 3.1 ^b^	38.6 ± 8.2 ^a^	9.8 ± 1.5 ^b^	76.3 ± 1.6 ^c^	54.5 ± 2.2 ^a^	12.3 ± 1.3 ^b^	133.2 ± 6.0 ^c^
arg	62.1 ± 1.7 ^a^*	43.3 ± 4.5 ^b^	10.0 ± 2.5 ^b^*	48.9 ± 10.4 ^a^	11.0 ± 1.9 ^b^	164.6 ± 1.6 ^c^	58.0 ± 2.9 ^a^	6.6 ± 0.1 ^b^	7.9 ± 0.7 ^b^
pro	286.5 ± 22.9 ^a^	58.84 ± 0.01 ^b^	269.7 ± 19.9 ^a^	183.1 ± 38.5 ^a^	23.6 ± 0.5 ^b^	146.9 ± 27.6 ^a^	212.5 ± 16.6 ^a^*	27.9 ± 4.8 ^b^	135.8 ± 22.5 ^c^*
cys	11.5 ± 1.1 ^a^	3.3 ± 1.1 ^a^	9.0 ± 3.1 ^a^	12.2 ± 1.6 ^a^	6.5 ± 4.4 ^a^	34.0 ± 2.7 ^b^	13.9 ± 2.9 ^a^*	3.5 ± 0.1 ^b^*	45.3 ± 7.8 ^c^
tyr	21.6 ± 0.7 ^a^*	6.9 ± 9.8 ^b^*	10.0 ± 2.5 ^b^*	17.1 ± 3.5 ^a^	12.3 ± 2.3 ^a^	8.1 ± 0.1 ^a^	19.9 ± 2.3 ^a^*	10.5 ± 1.0 ^ab^	8.7 ± 0.1 ^b^*
val	24.3 ± 2.4 ^a^	16.0 ± 5.2 ^a^	9.9 ± 0.7 ^a^	28.9 ± 10.7 ^a^*	8.9 ± 2.7 ^b^*	66.4 ± 0.4 ^c^	45.9 ± 7.8 ^a^	13.0 ± 3.0 ^b^	121.9 ± 9.3 ^c^
met	4.6 ± 1.8 ^a^	6.5 ± 1.9 ^a^	2.5 ± 0.3 ^a^	5.8 ± 2.5 ^a^	3.7 ± 1.5 ^a^	29.0 ± 1.2 ^b^	9.3 ± 2.4 ^a^	5.1 ± 1.6 ^a^	51.2 ± 2.1 ^b^
lys	20.5 ± 7.3 ^a^	8.9 ± 0.4 ^a^	5.8 ± 2.3 ^a^	23.5 ± 12.3 ^a^*	4.3 ± 0.2 ^b^*	81.4 ± 1.5 ^c^	48.8 ± 4.3 ^a^	9.0 ± 0.2 ^b^	108.9 ± 10.8 ^c^
ile	9.7 ± 13.7 ^a^	10.4 ± 0.7 ^a^	5.0 ± 2.9 ^a^	17.3 ± 7.4 ^a^	1.6 ± 1.1 ^a^	45.9 ± 0.7 ^b^	21.2 ± 10.8 ^a^	4.1 ± 0.9 ^a^	88.0 ± 7.0 ^b^
leu	8.4 ± 4.8 ^a^	7.4 ± 0.3 ^a^	0.0 ± 0.0 ^a^	19.5 ± 5.4 ^a^	7.0 ± 0.1 ^a^	106.3 ± 2.5 ^b^	36.1 ± 7.9 ^a^	14.6 ± 0.3 ^b^	183.1 ± 10.1 ^c^
phe	14.1 ± 5.7 ^a^	6.7 ± 0.4 ^a^	3.3 ± 4.6 ^a^	10.3 ± 9.8 ^a^	11.5 ± 0.8 ^a^	83.7 ± 0.1 ^b^	15.4 ± 15.6 ^a^	14.8 ± 1.5 ^a^	131.0 ± 6.6 ^b^
trp	44.1 ± 23.8 ^a^	15.3 ± 21.6 ^a^	31.1 ± 19.3 ^a^	26.1 ± 20.0 ^a^	18.0 ± 1.4 ^a^	21.1 ± 0.5 ^a^	20.1 ± 18.9 ^a^	14.1 ± 0.7 ^a^	27.9 ± 3.7 ^a^
TOTAL	956.2 ± 39.2 ^a^	468.0 ± 20.8 ^b^	592 ± 69.6 ^b^	745.4 ± 205.7 ^a^	200.4 ± 16.7 ^b^	1142.3 ± 13.4 ^c^	897.1 ± 110.4 ^a^	210.3 ± 9.8 ^b^	1415.6 ± 91.6 ^c^

Different lowercase letters within the same sample row indicate notable observed differences between treatments as determined by Dunn’s test with Bonferroni correction; these comparisons are reported for descriptive purposes only given the single-batch design. Asterisks (*) denote comparisons where 0.01 < *p* < 0.05; unmarked letter differences indicate *p* ≤ 0.01; (+) indicates amino acids for which the Scheirer–Ray–Hare non-parametric test was applied due to violation of normality assumptions; all other amino acids were analyzed by two-factor ANOVA. HPC: hemp press cake; OPC: oat press cake; RAW: untreated (H100, H75:O25, H50:O50); PAS: pasteurized (H100P, H75:O25P, H50:O50P); FER: fermented (H100F, H75:O25F, H50:O50F).

**Table A3:** Biogenic amine content (mg/kg sample) of hemp–oat press cake formulations across three processing treatments (RAW, PAS, FER). Values are expressed as mean ± standard deviation.

Sample	Blend Ratios		Treatment	Biogenic Amines (mg/kg)				
	HPC	OPC		Putrescine	Tyramine	Spermine	Spermidine	Total
H100	100	0	RAW	38.90 ± 0.51 ^a^*	12.58 ± 0.11 ^a^	51.88 ± 0.54 ^a^	233.04 ± 0.83 ^a^	336.40 ± 1.99 ^a^
H100P	100	0	PAS	29.32 ± 0.42 ^b^	30.40 ± 1.61 ^b^*	136.88 ± 0.99 ^b^	21.44 ± 0.57 ^b^	229.70 ± 2.93 ^b^
H100F	100	0	FER	37.43 ± 0.06 ^c^*	27.59 ± 0.96 ^c^*	38.8 ± 3.15 ^c^	194.92 ± 1.19 ^c^	298.82 ± 5.23 ^c^
H75:O25	75	25	RAW	26.40 ± 0.30 ^a^*	23.37 ± 0.14 ^a^	14.07 ± 0.38 ^a^*	93.89 ± 1.12 ^a^	157.73 ± 1.33 ^a^
H75:O25P	75	25	PAS	28.17 ± 0.94 ^b^*	36.39 ± 0.51 ^b^	80.45 ± 2.28 ^b^	4.47 ± 1.18 ^b^	152.81 ± 2.36 ^a^
H75:O25F	75	25	FER	32.18 ± 0.10 ^c^	24.90 ± 0.28 ^a^	19.50 ± 0.70 ^c^*	98.61 ± 0.74 ^c^	175.18 ± 1.26 ^b^
H50:O50	50	50	RAW	20.39 ± 0.09 ^a^	19.06 ± 0.04 ^a^	7.40 ± 0.05 ^a^	46.47 ± 0.20 ^a^	93.31 ± 0.13 ^a^
H50:O50P	50	50	PAS	22.45 ± 0.27 ^b^	33.44 ± 0.27 ^b^	47.93 ± 0.72 ^b^	5.79 ± 0.56 ^b^	113.18 ± 0.13 ^b^
H50:O50F	50	50	FER	24.52 ± 0.59 ^c^	15.49 ± 0.06 ^c^	5.59 ± 0.10 ^a^	27.85 ± 0.43 ^c^	73.46 ± 0.12 ^c^

Different lowercase letters within the same sample row indicate notable observed differences between treatments as determined by Tukey’s HSD post hoc test; these comparisons are reported for descriptive purposes only given the single-batch design. Asterisks (*) denote comparisons where 0.01 < *p* < 0.05; unmarked letter differences indicate *p* ≤ 0.01. All biogenic amines met normality assumptions and were analyzed by two-factor ANOVA; no non-parametric substitution was required. Histamine and cadaverine were excluded due to near-zero concentrations across all samples. HPC: hemp press cake; OPC: oat press cake; RAW: untreated (H100, H75:O25, H50:O50); PAS: pasteurized (H100P, H75:O25P, H50:O50P); FER: fermented (H100F, H75:O25F, H50:O50F).

**Table A4:** Protein carbonyl content (nmol carbonyl/mg protein) of hemp–oat press cake formulations across three processing treatments (RAW, PAS, FER). Values are expressed as mean ± standard deviation.

Sample	Blend Ratios		Treatment	Carbonyl Content (nmol Carbonyl/mg Protein)
	HPC	OPC		
H100	100	0	RAW	40.0 ± 8.9 ^a^
H100P	100	0	PAS	49.3 ± 12.3 ^a^
H100F	100	0	FER	38.5 ± 4.0 ^a^
H75:O25	75	25	RAW	33.7 ± 3.3 ^a^*
H75:O25P	75	25	PAS	26.9 ± 0.9 ^ab^
H75:O25F	75	25	FER	20.1 ± 0.6 ^b^*
H50:O50	50	50	RAW	35.3 ± 4.2 ^a^*
H50:O50P	50	50	PAS	27.5 ± 2.2 ^ab^
H50:O50F	50	50	FER	21.9 ± 2.2 ^b^*

Different lowercase letters within the same formulation group (H100, H75:O25, H50:O50) indicate notable observed differences between treatments as determined by Tukey’s HSD post hoc test; these comparisons are reported for descriptive purposes only given the single-batch design. Asterisks (*) denote comparisons where 0.01 < *p* < 0.05; unmarked letter differences indicate *p* ≤ 0.01. Statistical analysis was performed using two-factor ANOVA with formulation and treatment as fixed factors. HPC: hemp press cake; OPC: oat press cake; RAW: untreated; PAS: pasteurized; FER: fermented.

**Table A5:** Protein solubility (g/100 g protein) of hemp–oat press cake formulations across three processing treatments (RAW, PAS, FER), determined under eight solvent conditions targeting specific protein interaction forces: native state protein (NSP), hydrogen bonds (HBs), disulfide bonds (DISBs), hydrophobic interactions (HYDXs), and their combinations. Values are expressed as mean ± standard deviation.

Sample	Blend Ratios		Treatment	Protein Solubility (g/100 g Protein)							
	HPC	OPC		NSP	HB	DISB	HYDX	HB × DISB	HB × HYDX	DISB × HYDX	HB × DISB × HYDX
H100	100	0	RAW	12.6 ± 0.9 ^a^	39.4 ± 5.1 ^a^	11.2 ± 1.1 ^a^	37.2 ± 1.3 ^a^	42.7 ± 2.1 ^a^	34.7 ± 0.5 ^a^	59.3 ± 1.1 ^a^	66.6 ± 0.2 ^a^
H100P	100	0	PAS	5.6 ± 0.3 ^b^	22.0 ± 1.6 ^b^	6.1 ± 0.3 ^b^*	5.7 ± 0.5 ^b^	39.1 ± 1.6 ^a^	48.2 ± 0.4 ^b^	32.4 ± 1.2 ^b^	64.2 ± 1.5 ^ab^
H100F	100	0	FER	5.0 ± 0.2 ^b^	17.5 ± 1.3 ^b^	4.4 ± 0.4 ^c^*	5.0 ± 0.7 ^b^	42.1 ± 4.9 ^a^	45.6 ± 0.7 ^b^	36.8 ± 1.2 ^c^	61.5 ± 2.1 ^b^
H75:O25	75	25	RAW	11.9 ± 1.0 ^a^	36.5 ± 0.5 ^a^	11.2 ± 0.6 ^a^	23.0 ± 1.6 ^a^	48.0 ± 2.8 ^a^	33.7 ± 0.5 ^a^	45.4 ± 1.3 ^a^	45.5 ± 0.6 ^a^
H75:O25P	75	25	PAS	3.2 ± 0.6 ^b^	25.5 ± 1.9 ^b^	3.9 ± 0.1 ^b^	15.2 ± 1.7 ^b^	35.2 ± 2.2 ^b^	23.5 ± 0.5 ^b^	46.5 ± 1.3 ^a^	48.2 ± 1.9 ^a^
H75:O25F	75	25	FER	4.6 ± 0.9 ^b^	16.8 ± 1.3 ^c^	5.1 ± 1.6 ^b^	9.8 ± 0.9 ^c^	35.2 ± 2.5 ^b^	33.9 ± 0.7 ^a^	37.9 ± 3.0 ^b^	46.3 ± 0.1 ^a^
H50:O50	50	50	RAW	14.5 ± 1.3 ^a^	42.3 ± 0.6 ^a^	15.4 ± 0.8 ^a^	26.9 ± 1.9 ^a^	70.1 ± 1.4 ^a^*	51.3 ± 1.5 ^a^	52.5 ± 1.7 ^a^	60.8 ± 0.8 ^a^
H50:O50P	50	50	PAS	0.7 ± 1.2 ^b^	17.2 ± 1.0 ^b^	10.3 ± 0.5 ^b^	32.7 ± 0.9 ^b^	63.6 ± 3.6 ^b^*	37.3 ± 0.1 ^b^	69.2 ± 1.2 ^b^	55.9 ± 1.5 ^b^*
H50:O50F	50	50	FER	5.3 ± 1.7 ^c^	27.5 ± 1.1 ^c^	5.4 ± 0.1 ^c^	12.8 ± 1.2 ^c^	41.7 ± 3.0 ^c^	33.3 ± 3.5 ^c^	46.1 ± 1.0 ^c^	59.8 ± 2.2 ^a^*

Different lowercase letters within the same formulation group (H100, H75:O25, H50:O50) indicate notable observed differences between treatments as determined by Tukey’s HSD post hoc test; these comparisons are reported for descriptive purposes only given the single-batch design. Asterisks (*) denote comparisons where 0.01 < *p* < 0.05; unmarked letter differences indicate *p* ≤ 0.01. Statistical analysis was performed using two-factor ANOVA with formulation and treatment as fixed factors. Solvent conditions are cumulative: combination columns reflect the simultaneous disruption of the indicated interaction forces. HPC: hemp press cake; OPC: oat press cake; RAW: untreated; PAS: pasteurized; FER: fermented.

**Table A6:** Phytic acid content (g/100 g sample) of hemp–oat press cake formulations across three processing treatments (RAW, PAS, FER). Values are expressed as mean ± standard deviation.

Sample	Blend Ratios		Treatment	Phytic Acid Content (g/100 g Sample)
	HPC	OPC		
H100	100	0	RAW	5.39 ± 0.18 ^a^
H100P	100	0	PAS	5.35 ± 0.03 ^a^
H100F	100	0	FER	5.47 ± 0.05 ^a^
H75:O25	75	25	RAW	4.17 ± 0.06 ^a^
H75:O25P	75	25	PAS	4.17 ± 0.05 ^a^
H75:O25F	75	25	FER	4.20 ± 0.05 ^a^
H50:O50	50	50	RAW	3.27 ± 0.02 ^a^
H50:O50P	50	50	PAS	4.17 ± 0.06 ^b^
H50:O50F	50	50	FER	3.23 ± 0.04 ^a^

Different lowercase letters within the same formulation group (H100, H75:O25, H50:O50) indicate notable observed differences between treatments as determined by Tukey’s HSD post hoc test; these comparisons are reported for descriptive purposes only given the single-batch design. No asterisks appear in this table, as no comparisons fell in the range 0.01 < *p* < 0.05. Statistical analysis was performed using two-factor ANOVA with formulation and treatment as fixed factors. HPC: hemp press cake; OPC: oat press cake; RAW: untreated; PAS: pasteurized; FER: fermented.

## Appendix B. Additional Statistical Analysis Results: PCA, ANOVA

**Table A7:** Total amino acid PCA. Summary of principal component analysis (PCA) results for total amino acid content, including percentage of variance explained, cumulative variance and contributions of individual amino acids to the first two principal components (PC1 and PC2).

Principal Component		PC1	PC2
% of variance		59.854	19.950
Cumulative % of variance		59.854	79.804
Variable contributions (%)	asx	**8.604**	2.133
	glx	**7.841**	4.464
	Serine	**9.027**	1.586
	Glycine	**8.065**	0.349
	Histidine	0.012	**26.721**
	Threonine	5.017	4.461
	Alanine	**7.629**	3.462
	Arginine	**9.331**	0.229
	Proline	0.926	0.807
	Cysteine	0.209	**25.155**
	Tyrosine	5.482	3.222
	Valine	7.998	2.907
	Methionine	1.068	**12.352**
	Lysine	**6.383**	0.001
	Isoleucine	**6.048**	7.821
	Leucine	**8.468**	0.563
	Phenylalanine	**7.893**	3.766

asx = aspartic acid + asparagine; glx = glutamic acid + glutamine. Main contributor variables for each principal component are highlighted in bold. Only the first two principal components are shown, as they explain approximately 80% of total variance.

**Table A8:** Free amino acid PCA. Summary of principal component analysis (PCA) results for free amino acid content, including percentage of variance explained, cumulative variance and contributions of individual amino acids to the first two principal components (PC1 and PC2).

Principal Component		PC1	PC2
% of variance		63.403	18.742
Cumulative % of variance		63.403	82.145
Variable contributions (%)	Aspartic acid	2.262	**16.370**
	Asparagine	4.900	7.666
	Glutamic acid	0.590	**14.777**
	Serine	6.720	1.171
	Glycine	**7.777**	0.439
	Histidine	5.430	5.027
	Threonine	**7.883**	0.199
	Alanine	**7.695**	0.742
	Arginine	0.595	3.744
	Proline	0.118	**22.809**
	Cysteine	7.715	0.000
	Tyrosine	0.456	**14.664**
	Valine	**8.010**	0.000
	Methionine	**7.938**	0.906
	Lysine	**7.903**	0.026
	Isoleucine	**8.077**	0.080
	Leucine	**7.974**	0.745
	Phenylalanine	**7.736**	0.941
	Tryptophan	0.223	**9.692**

Main contributor variables for each principal component are highlighted in bold. Only the first two principal components are shown, as they explain more than 80% of total variance.

**Table A9:** Biogenic amine PCA. Summary of principal component analysis (PCA) results for four biogenic amine contents, including percentage of variance explained, cumulative variance, and contributions of individual amines to the first two principal components (PC1 and PC2).

Principal Component	% of Variance	Cumulative % of Variance	Variable Contributions (%)			
			Putrescine	Tyramine	Spermine	Spermidine
PC1	52.723	52.723	**26.813**	24.100	5.867	**43.220**
PC2	36.345	89.068	**27.615**	19.440	**49.864**	3.080

The main contributor variables for each principal component are highlighted in bold. Only the first two principal components are shown, as they explain >80% of total variance.

**Table A10:** Protein oxidation two-factor ANOVA results. Summary of two-factor ANOVA results for protein oxidation.

Factor	F	*p*-Value	Effect Size (Ges)
Formulation	21.509	<0.001	0.705
Treatment	7.187	0.005	0.444
Interaction	2.295	0.099	0.338
Simple effects of treatment within each formulation			
H100	3.178	0.066	0.261
H75:O25	4.343	0.029	0.326
H50:O50	4.255	0.031	0.321
Simple effects of formulation within each treatment			
RAW	1.03	0.377	0.103
PAS	15.294	<0.001	0.63
FER	9.775	0.001	0.521

**Table A11:** Protein solubility two-factor ANOVA results. Summary of two-factor ANOVA results for protein solubility using eight different bond disrupting solutions.

Solution	Disrupted Bonds	Factor	F-Value	*p*-Value	Effect Size (Ges)
1	NSP	Formulation	3.349	0.058	0.271
		Treatment	246.838	<0.001	0.965
		Interaction	10.361	<0.001	0.697
2	HB	Formulation	5.213	0.016	0.367
		Treatment	234.182	<0.001	0.963
		Interaction	18.830	<0.001	0.807
3	DISB	Formulation	61.831	<0.001	0.873
		Treatment	246.244	<0.001	0.965
		Interaction	13.065	<0.001	0.744
4	HYDX	Formulation	124.787	<0.001	0.933
		Treatment	555.564	<0.001	0.984
		Interaction	179.363	<0.001	0.976
5	HB × DISB	Formulation	119.761	<0.001	0.930
		Treatment	52.959	<0.001	0.855
		Interaction	24.426	<0.001	0.844
6	HB × HYDX	Formulation	219.995	<0.001	0.961
		Treatment	16.325	<0.001	0.645
		Interaction	137.734	<0.001	0.968
7	DISB × HYDX	Formulation	209.026	<0.001	0.959
		Treatment	150.422	<0.001	0.944
		Interaction	158.605	<0.001	0.972
8	HB × DISB × HYDX	Formulation	349.425	<0.001	0.975
		Treatment	4.183	0.032	0.317
		Interaction	8.897	<0.001	0.664

**Table A12:** Phytic acid two-factor ANOVA. Summary of two-factor ANOVA results for phytic acid content.

Factor	F	*p*-Value	Effect Size (Ges)
Formulation	1423.311	<0.001	0.994
Treatment	39.594	<0.001	0.815
Interaction	56.182	<0.001	0.926
Simple effects of treatment within each formulation			
H100	1.892	0.18	0.174
H75:O25	0.169	0.845	0.018
H50:O50	149.898	<0.001	0.943
Simple effects of formulation within each treatment			
RAW	609.807	<0.001	0.985
PAS	251.371	<0.001	0.965
FER	674.498	<0.001	0.987

**Table A13:** Water-holding capacity Welch ANOVA. Summary of Welch-ANOVA results for water-holding capacity.

Parameter	N	Statistic	*p*	Method
WHC	45	345.29	2.15 × 10^−10^	Welch ANOVA

**Table A14:** Oil-holding capacity two-factor ANOVA. Summary of two-factor ANOVA results for oil-holding capacity.

Factor	F	*p*-Value	Effect Size (Ges)
Formulation	82.995	<0.001	0.822
Treatment	41.54	<0.001	0.698
Interaction	8.778	<0.001	0.494
Simple effects of treatment within each formulation			
H100	40.657	<0.001	0.693
H75:O25	10.802	<0.001	0.375
H50:O50	7.638	0.002	0.298
Simple effects of formulation within each treatment			
RAW	14.473	<0.001	0.446
PAS	5.548	0.008	0.236
FER	80.53	<0.001	0.817
